# Circularizing PET-G Multimaterials: Life Cycle Assessment and Techno-Economic Analysis

**DOI:** 10.1021/acssuschemeng.3c04047

**Published:** 2023-10-13

**Authors:** Peng Huang, Ashiq Ahamed, Ruitao Sun, Guilhem X. De Hoe, Joe Pitcher, Alan Mushing, Fernando Lourenço, Michael P. Shaver

**Affiliations:** † Department of Materials, Henry Royce Institute, The University of Manchester, Manchester M13 9PL, U.K.; ‡ Pragmatic Semiconductor Ltd., Cambridge CB4 0WH, U.K.; § School of Engineering, The University of Manchester, Manchester M13 9PL, U.K.; ∥ Mastercard DigiSec Lab, 5 Booths Park, Chelford Road, Knutsford WA16 8QZ, U.K.

**Keywords:** life cycle assessment, techno-economic analysis, chemical recycling, multimaterials, PET-G

## Abstract

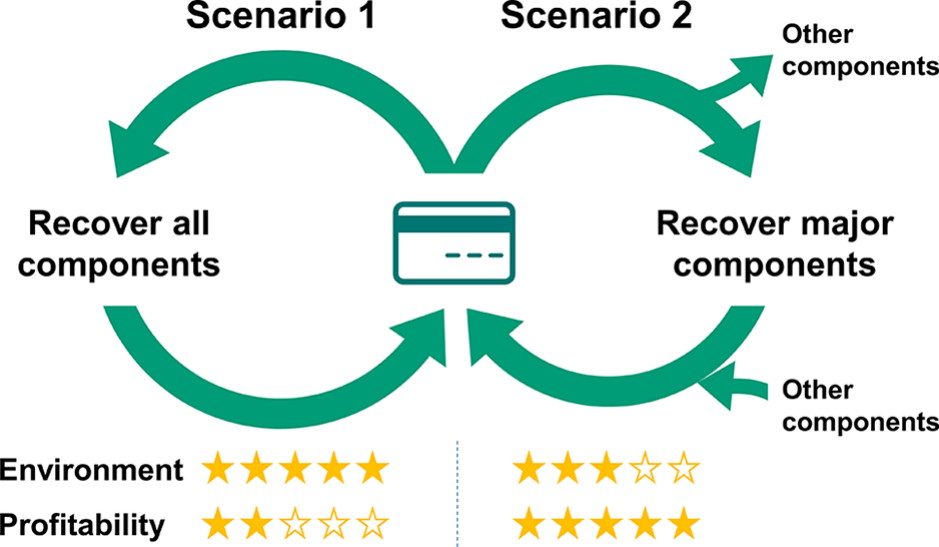

The recycling of multimaterials such as payment or access cards poses significant challenges. Building on previous experimental work demonstrating the feasibility of chemically recyclable payment cards made from glycol-modified poly(ethylene terephthalate) (PET-G), we use life cycle assessment and techno-economic analysis to investigate two chemical recycling scenarios and evaluate their potential environmental and economic benefits. Recovering all components from the depolymerized products (Scenario 1) achieves substantial environmental benefits across most categories, reducing global warming by up to 67% compared to only recovering major components (Scenario 2). However, the environmental benefits in Scenario 1 incur 69% higher total annualized costs, causing its profitability to be dependent on a minimum selling price of £13.4/kg for cyclohexanedimethanol and less than a 10% discount rate. In contrast, Scenario 2 is less sensitive to discount rate variation and thus a lower risk and more economically feasible option, albeit less environmentally sustainable.

## Abstract 2

Recovering all components from payment cards yields greater environmental benefits, whereas recovering major components is more economically feasible.

## Introduction

The annual production of plastic has witnessed a staggering surge from a mere 2 million tons in the 1950s to an estimated 367 million tons in 2020.^1^ Unfortunately, over 90% of plastic products end up in landfills, incinerators, or the environment,^2^ making plastic a symbol of the unsustainable “take-make-dispose” linear economy.^3^ The extensive consumption and unmanaged disposal of end-of-life plastic products come at significant environmental and economic costs^4^ due to the slow natural degradation of petrochemical products^5^ and a lack of functional waste management infrastructure. Fortunately, a circular economy model presents a promising pathway toward an environmentally sustainable future for plastics.^6^ The model advocates for retaining materials in their highest value condition throughout the life cycle, thus reducing waste and promoting sustainability.^7^ To achieve this vision, it is urgent to address the prevailing challenges of plastic complexity and diversity^8^ and develop economically feasible solutions for recycling waste plastics.^9^ This will not only ensure sustainability but also reduce environmental and economic costs. In many instances, a holistic approach that encompasses the redesign of plastics,^10^ greater transparency and coordination along supply chains, suitable waste management,^11^ and strong regulatory intervention is essential to attain true circularity.^12^

While mechanical recycling is often perceived as the most economically (i.e., less energy-intensive) and environmentally (e.g., less greenhouse gas release) viable option for expanding the life cycle boundaries of plastic monomaterials, this method suffers from drawbacks such as a finite number of recycling cycles,^13^ poor materials retention, and a reduction in molecular weight during reprocessing, especially when materials are not appropriately sorted.^14^ In contrast, chemical depolymerization can prolong the lifespan limits of plastics by utilizing the end products as building blocks for manufacturing virgin-quality polymers,^15^ thereby conserving energy, reducing reliance on nonrenewable fossil resources, and enabling greater adaptability to market demands.^16^ Recent developments in catalytic systems for solvolysis reactions have sparked interest in this selective chemical recycling methodology and facilitated rapid progress in technology that permits milder reaction conditions, improved energy efficiency,^17^ high tolerance of contamination with unknown chemicals (e.g., additives and fillers), and effective handling of multimaterials. However, the integration of environmental concerns and economic considerations related to manufacturing, infrastructure, markets, and trade is crucial in shaping the direction of these pathways.^18^

Life cycle assessment (LCA) is an effective tool for weighing the environmental and energy consequences of different approaches to managing plastic waste,^19^ including landfill, recycling, composting, and energy recovery.^20^ By assessing the environmental performance of these end-of-life options, LCA enables ranking and decision-making^21^ based on the most environmentally sustainable and eco-friendly choices.^22^ Our previous work demonstrated the feasibility of chemical recycling of multimaterial cards (i.e., those used for payment or access) composed of glycol-modified poly(ethylene terephthalate) (PET-G) laminated sheets interwoven with diverse metals and materials in the antennae, chips, magnetic stripes, and holograms.^23^ For the chemical recycling of polyesters, tools such as glycolysis,^24,25^ hydrolysis,^26^ aminolysis,^27^ and methanolysis are necessary for innovation.^28,29^ The logical progression of this proof-of-concept work is to probe the potential environmental and economic impacts of the chemical recycling process to enable plastic card circularity.

In this study, we undertake a rigorous and comprehensive modeling effort for the chemical recycling of PET-G plastic cards into constituent monomers and metal components, encompassing all utilities required for an integrated process. We employed LCA to estimate the various environmental impacts and identify the hotspots in the process. Our comparable tools consider the environmental and economic impacts associated with the chemical recycling of multilayered PET-G cards, unpicking the critical drivers for realizing the depolymerization of plastic cards at scale. These tools also facilitate the sharing of traceable data and promote transparency, trust, and accountability in decision-making in terms of technological development, infrastructure investment, and policy development. We have outlined the key steps in the recycling process and identified significant sustainability factors that can lead to reduced byproduct emissions and resource consumption. Additionally, we have conducted a techno-economic analysis (TEA) to predict the capital and operating costs, including the sale of the depolymerized product. Sensitivity analysis was employed to highlight the relative importance of the process variables that can be modified for further process improvement and optimization.

## Material and Methods

### Life Cycle Assessment

#### LCA Methodology

This study adhered to the international standards of “ISO 14040: Principles and Framework” and “ISO 14044: Requirements and Guidelines” to develop the LCA model.^30^ The goal of the study is to assess and measure the environmental impacts resulting from (a) the process of depolymerizing waste PET-G plastic cards (supplied by Mastercard, compositions provided in Figure S3), (b) separating and purifying the depolymerized products, and (c) reclaiming solvents. The scope of this investigation was to perform a “system” LCA encompassing all unit operations, which began with the depolymerization process and ended at the point when the recycled products were recovered from the system. Materials that were not recoverable at any processing step, such as fillers and additives, were assumed to be disposed of within the wastewater once they left the system. The study did not consider the production and use of payment cards before they become waste. The functional unit was defined as the treatment of 1 tonne of PET-G payment cards per day, and feedstock material properties are presented in Table 1. A cradle-to-gate system boundary and cutoff approach were used in the LCA, starting at the gate of the waste management facility. A comparative LCA was carried out using SimaPro software (V9.4, PRé Sustainability B.V.) with the ecoinvent database (V3.9). The evaluation of the entire process’ impacts on the environment followed the Hierarchist cultural perspective, which represents the scientific model consensus for a century-spanning time frame, in accordance with ISO 14044.^31^ The study employed a hotspot analysis to identify significant emission sources in the overall process, highlighting areas of concern. Furthermore, sensitivity analysis was performed to investigate the environmental impact range when using different amounts of water in the purification process.

**Table 1:** Resource Input, Output, and Energy Consumption for the Foreground Processes^a^

product/process	S1 (tonne FU^–1^)	S2 (tonne FU^–1^)
input		
EG	0.542	1.996
organocatalyst	0.035	0.035
payment cards	1	1
water	0.3027	3
acetone	0.0009	0.0009
output		
BHET	0.9473	0.9435
CHDM	0.1836	
metals	0.0055	0.0055
wastewater	0.744	4.3811
energy consumption (electricity, kJ)		
depolymerization	8.01 × 10^5^	8.01 × 10^5^
distillation of acetone	191.34	191.34
evaporation of water	3.00 × 10^7^	
distillation of EG	1.54 × 10^6^	
distillation of CHDM	7.73 × 10^4^	

a Note: S1, Scenario 1; S2, Scenario 2; FU, functional unit.

### Life Cycle Modeling and Inventory

The LCA model was constructed by using experimental findings. In cases where data were unavailable, secondary sources such as Aspen Plus modeling data, literature, patents, ecoinvent database, and commercial sources were acquired based on the principle of best fit. The laboratory-scale experiments provided the mass-balance data, which were then extended to one functional unit (FU).

**Figure 1:**
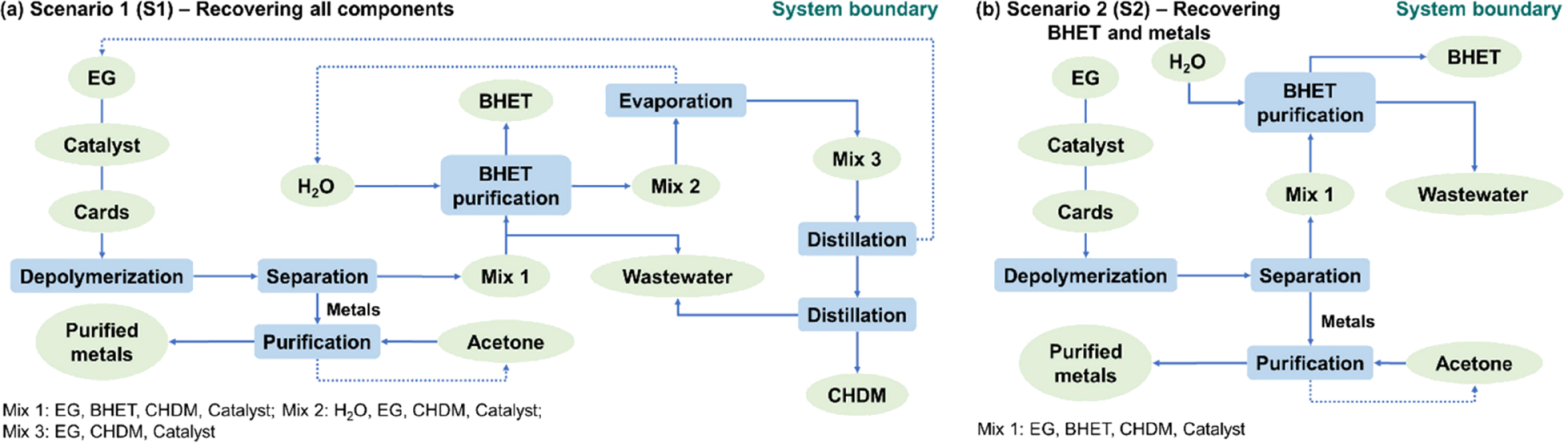
Process flow diagrams show the system boundary of the foreground process, background process, and materials flow in this work. (a) Recover all of the depolymerized products. (b) Recover only metals and BHET, disposing of the other components as wastewater. Cards: made of PET-G. Catalyst: DBU. Solid arrows represent process flow and dotted arrows indicate recycling.

Two scenarios were investigated in the LCA modeling. Figure 1 depicts the process flow diagram and the associated system boundary, which encompasses the foreground processes of plastic card depolymerization, product separation, and purification. The background processes include the synthesis of organocatalyst, bis(2-hydroxyethyl) terephthalate (BHET), and 1,4-cyclohexanedimethanol (CHDM). The diagram also illustrates the flow of materials in the system. Scenario 1 (Figure 1a) mainly includes five processing steps: (1) depolymerization, where PET-G payment cards are depolymerized in a mixed solution of ethylene glycol (EG) and organocatalyst (1,8-diazabicyclo [5.4.0] undec-7-ene, DBU); (2) separation, where the metals are separated from the colloidal suspension; (3) BHET recovery, where hot deionized water (∼80 °C) is added to the suspension, followed by filtration and cooling to 2 °C to afford a white precipitate. The mixture is then filtered to obtain BHET. (4) Solvent recovery, where water and EG are recovered by distillation and recycled back for reuse. (5) CHDM recovery, where CHDM is recovered by distillation. On the other hand, Scenario 2 considers only the recovery of the metals and BHET from the depolymerized mixture; the remaining components are disposed of as wastewater (Figure 1b).

Tables S2 and S3 present the mass balance of chemicals and products for the key background processes: synthesis of BHET, CHDM, and DBU (see associated Schemes S1–S4). In all cases, a 5% weight loss was assumed during sample preparation due to the multiple steps involved. The ecoinvent database was used to obtain the data for common chemicals; however, since no database information was available for the hydroxylamine sulfate needed for DBU synthesis, hydroxylamine was used instead. HNO_3_ solution, ammonium solution, and sulfuric acid were excluded in the emissions in the synthesis of CHDM and DBU as materials can be recovered. However, air emissions that occurred during the two processes were taken into account. The end-of-life scenario for the Pd/C catalyst was excluded from the model, assuming that the catalyst can be regenerated for reuse.^32^ In the LCA model, the potential environmental benefits of the Pd/C catalyst (associated with the recovery of CHDM) were not claimed.

Table 1 presents the inventory of the foreground processes, comprising depolymerization, separation, and product purification, concerning product and material waste. The composition of PET-G cards was determined by weighing individual components and thermogravimetric analysis (Figures S1 and S2). In the absence of an inventory database for BHET, CHDM, and DBU, the published literature^33,34^ and patents^35^ were consulted for data related to the synthesis of these chemicals. The inventory data for EG, metals (scrap copper), acetone, and water were sourced from the ecoinvent database.

The energy consumption for the foreground processes was modeled using the Aspen Plus software based on calculated material usage. The electricity mix data set for Great Britain from ecoinvent was adopted for the electricity supply for all the processes. Life cycle inventory (LCI) analysis was conducted to assess and quantify the materials, resources, and emissions linked to the various stages of the system.

### Impact Categories

The environmental impacts were assessed using the ReCiPe 2016 Midpoint impact assessment method across categories such as global warming, human carcinogenic and noncarcinogenic toxicities, mineral and fossil resource scarcities, marine, freshwater, and terrestrial ecotoxicities, marine and freshwater eutrophications, terrestrial acidification, ionizing radiation (IR), ozone formations, fine particulate matter formation, stratospheric ozone depletion, land use (LU), and water consumption.

### Scenario Description

The present study analyzed the environmental impacts of the chemical recycling of PET-G-based multimaterial cards by comparing two distinct scenarios. Scenario 1 involved the recovery of all depolymerized products, while in Scenario 2, only metals and BHET were recovered, and the remaining components were disposed as wastewater. Byproducts such as additives and fillers were assumed to be disposed as wastewater in both scenarios. The analysis employed a system expansion approach whereby the recovered products were credited with offsetting their respective production from virgin materials. The study excluded the impacts associated with infrastructure (e.g., reactor, columns, filter, coolers, mixers, pumps) in the analysis. As there is an opportunity to modify the amount of water used during BHET purification, a sensitivity analysis was performed to understand the influence of this parameter on the overall chemical recycling process via several environmental metrics: energy consumption, greenhouse gas emission, toxicity, LU, and water use.

### Techno-Economic Analysis

#### Techno-Economic Analysis Modeling

The process simulation of separation and purification after chemical depolymerization was conducted using Aspen Plus (V12.1, Aspen Technology Inc., USA) on a daily basis of 1 tonne of cards. The nonrandom two-liquid Redlich–Kwong (NRTL-RK) method was utilized to calculate the thermodynamic properties of the multicomponent system. Missing physical properties were obtained from either the NIST Thermo Data Engine (TDE) or estimated using the Aspen Plus Property Constant Estimation System (PCES).^36^ As the conversion rate of the polymer had been determined previously through experiments, the scope of this simulation was limited to separation and purification (i.e., postdepolymerization processes). Comprehensive details pertaining to the depolymerization reaction, encompassing operational parameters, specific reactions, and resulting yields are extensively documented in our preceding publication.^23^ This information was employed to determine the feed composition in the present study. The recycled products were obtained through a series of downstream processing steps: separation, low-temperature crystallization, and distillation. The base case scenario involved a cooler, filter, single-stage solids washer (SWash), two-outlet flash (Flash2), mixer, separator, and distillation column. The filter was used to separate metals from the liquid fractions of the depolymerized products. The acetone evaporator was modeled as Flash2, and the distillation columns for water, EG, and CHDM were modeled using RadFrac. Additionally, SWash was incorporated to simulate the separation of washed metals from the solvent, and the separator was employed to isolate BHET crystals from the cooled liquid mixture.

### Economic Analysis Method

The Aspen Process Economic Analyzer (APEA, V12) was utilized to perform the economic analysis. Process models were employed to derive material and energy balances, which were then used to estimate the costs of raw materials and utilities, operating costs, product sales, and capital investment with detailed equipment sizing. Online databases were used to source consumable prices to facilitate the analysis. The simulation incorporated certain assumptions regarding the chemical recycling project in the UK, such as assuming a grass-roots project type that would commence in 2025. Additionally, an arbitrary 27-week duration for the Engineering, Procurement, and Construction (EPC) phase, as well as a 12-week start-up period, was factored into the simulation.

The separation and purification process costs for the two scenarios were estimated using APEA instead of relying on installation factors. APEA is advantageous in that it can calculate costs based on required materials and labor, and its combination of expert systems and mathematical models results in more precise economic measurements. The costs for the inputs and outputs used in the analyses and their respective sources are shown in Table S8.

## Results and Discussion

The primary challenges in plastic card recycling include securing a consistent and economical supply of feedstock (cards), optimizing the depolymerization process for efficiency, enhancing the downstream purification of monomers, and achieving a cost-effective balance between the scale of depolymerization and repolymerization processes. To achieve these goals, the end-of-life cards need to be collected by the local banks or partners under a responsible and trustworthy collection scheme and stored securely until a specific volume is reached. This process ensures the prevention of card material from being dropped in landfills. Initiatives that aim to enable cardholders to dispose of expired cards in a secure and sustainable manner hold the potential to pave the way for circularity and economics of scale in payment card recycling.^37^ After securing postconsumer feedstock via this proposed “closed-loop” collection scheme, the next practical challenge is to effectively separate the different types of cards according to the chemical identity of the plastic component, which is expected to be poly(vinyl chloride), poly(ethylene terephthalate), poly(lactic acid), and/or PET-G. This can be further complicated by the use of several plastics in one card (i.e., plastic laminates), a strategy often utilized in card fabrication to satisfy rigorous performance requirements. Simplifying the card design to a single plastic component would clearly aid in the card recycling process, but even within the current landscape of payment cards, it is possible to employ infrared spectroscopy combined with principal component analysis to effectively separate them into groups based on their plastic component(s) (Figures S4 and S5). Using this approach, it is clear that the PET-G and PET-based cards can be distinguished and sorted, thus forming the basis for a relatively clean feedstock for further chemical recycling into the constituent monomers (BHET and CDHM), whereas the metals (e.g., copper, gold, and palladium) can be recovered and remanufactured to enable a second life.

### Life Cycle Impact Assessment

**Figure 2:**
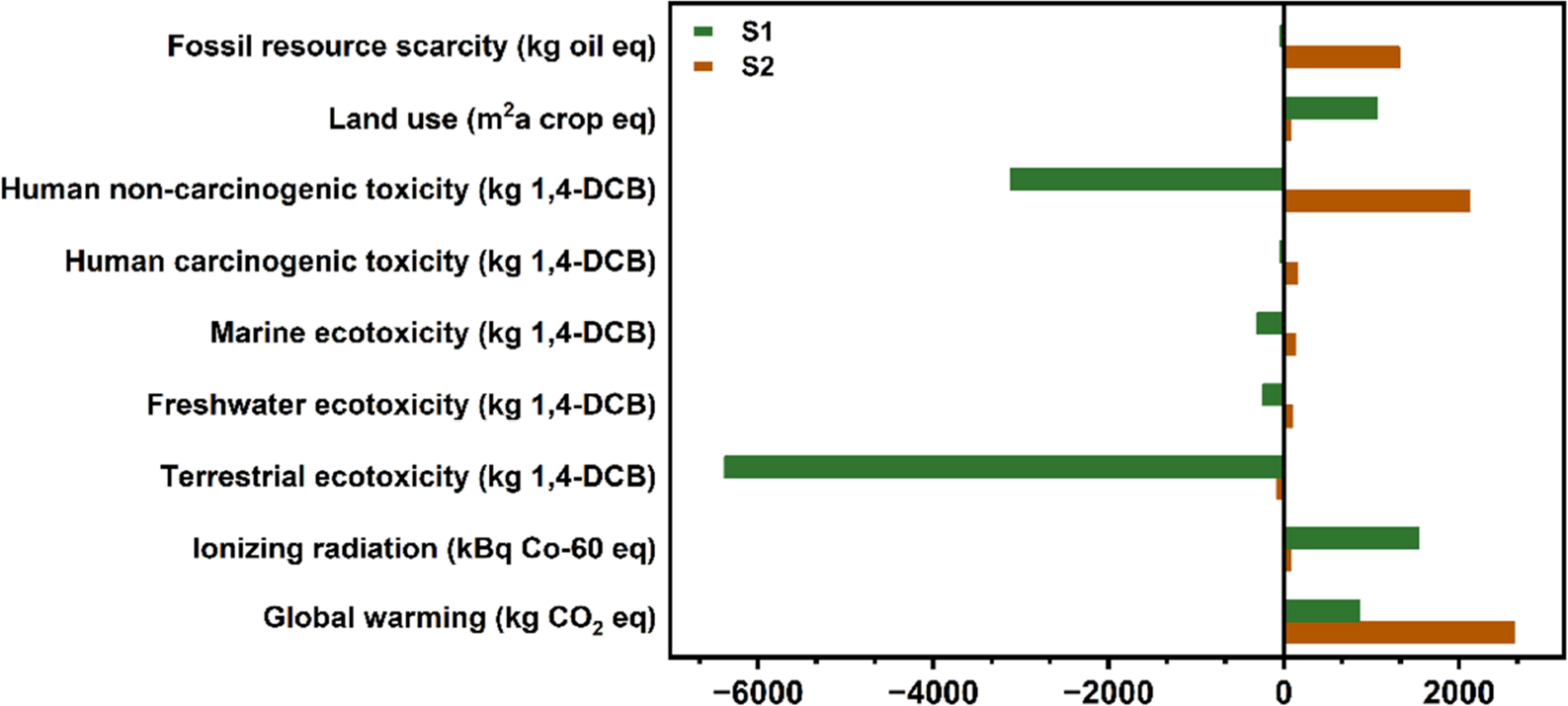
Overall environmental impact assessment of two end-of-life scenarios in the chemical recycling process. S1: recover all components. S2: recover only metals and BHET, with the remaining components disposed as wastewater. The results were obtained by using the ReCiPe midpoint (H) method.

We started the investigation of the environmental impacts associated with the chemical recycling of PET-G cards by comparing different end-of-life scenarios. From an economic perspective, it is tempting to recover only the major components—metals and BHET—from the payment cards and discard the rest as wastewater, given the considerable amount of energy needed to evaporate large amounts of water and distill the high boiling point EG solvent. However, we propose that recycling and reusing the solvents employed in the reaction and purification processes are more ecologically responsible approaches. To support this hypothesis, we designed two scenarios (S1 and S2) that encompass various degrees of product recovery. For both scenarios, we assessed the environmental impact of recovering different components from depolymerized products. Environmental benefits resulting from product displacement were denoted by negative numbers, whereas direct and indirect emissions are environmental burdens and thus represented by positive numbers (Figure 2). Both scenarios showed negligible impacts in the categories of eutrophication, ozone formation/depletion, acidification, respiratory matter, and mineral resource scarcity. When only BHET and metals are recovered (S2), we found that the only apparent benefit was for terrestrial ecotoxicity (TE) ( −78.4 kg 1,4-DCB), whereas the majority of impact categories demonstrated net negative effects, with global warming (2636.2 kg CO_2_ eq) being the most significant environmental burden, followed by human noncarcinogenic toxicity (HNCT) (2120.1 kg 1,4-DCB). In contrast, when the other main components (i.e., water, EG, and CHDM) were also recovered during chemical recycling (S1), we observed net positive environmental benefits in several categories, especially for TE and HNCT. Of the impact categories where both scenarios demonstrated environmental burdens, only two show S1 performing worse than S2: IR and LU. This is primarily due to the higher energy costs associated with the evaporation of water required in S1 (Table 1). In a recent publication, Selvam et al. conducted a study on PET glycolysis, exploring the global warming potential (GWP) using zinc oxide as the catalyst in a microwave-assisted approach, alongside homocatalysts. Their findings indicated GWP values in a similar order of magnitude (0.5–1 kgCO_2_eq/kg BHET) as those reported herein.^38^ Overall, the impact assessment highlights that designing a chemical recycling system that recovers EG and CHDM (i.e., S1) can significantly reduce global warming (∼67%), TE (∼8037%), and HNCT (∼247%).

**Figure 3:**
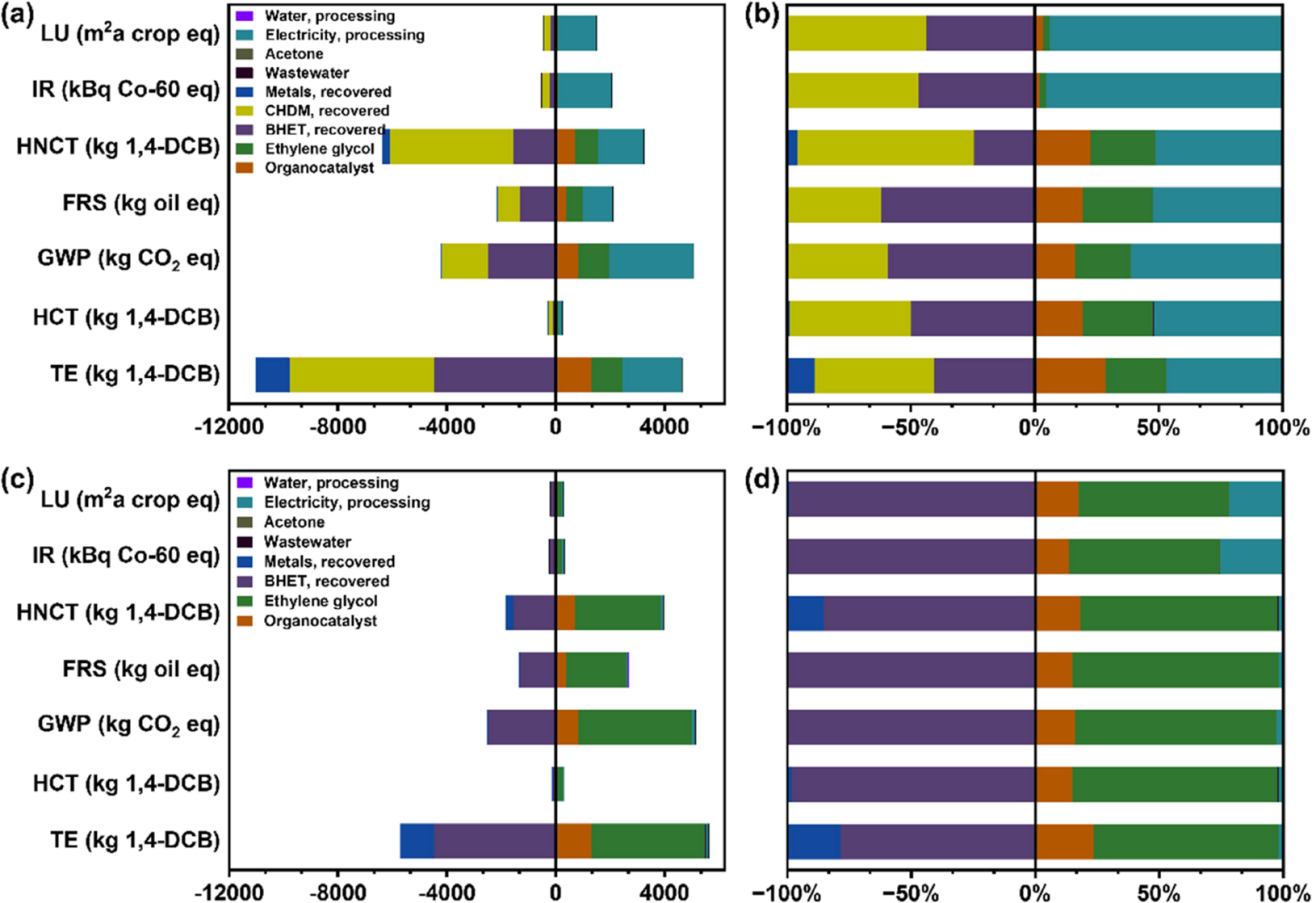
Comparison of the major environmental impacts of different scenarios. (a) Absolute values and (b) percentage of process contributions of S1. (c) Absolute values and (d) percentage of process contributions of S2. LU: land use; IR: ionizing radiation; HNCT: human noncarcinogenic toxicity; FRS: fossil resource scarcity; GWP: global warming potential; HCT: human carcinogenic toxicity; TE: terrestrial ecotoxicity; BHET: bis(2-hydroxyethyl) terephthalate; CHDM: 1,4-cyclohexanedimethanol.

To gain more insight into how the proposed chemical recycling processes affect the positive or negative impacts observed in certain categories, we performed a detailed contribution analysis for both scenarios (Figure 3). The aspects that increase impact are the use of EG, electricity, and catalysts. Despite the small amount of catalyst used (∼1 wt%), its environmental impacts are clearly significant. The absolute contributions of catalyst are the same for each impact category across both scenarios and are most pronounced for HNCT, fossil resource scarcity (FRS), GWP, and TE. However, the two chemical recycling scenarios employ many different amounts of EG and energy, as reflected in both the absolute and relative contributions to each impact category. In S2, the majority of the environmental burden (>60%) are caused by the large amount of EG needed, and the relative contributions of catalyst and electricity are small. In S1, the recovery of EG lowers its environmental burden, although there is still some positive contribution because not all the EG is recovered (some is consumed to produce BHET). With the reduction in EG, the large amounts of energy required to recover water, CHDM, and EG (Table 1) cause electricity to be the major positive contributor across the highlighted impact categories for S1. In most categories, the relative environmental burden from the catalyst was less than that from EG, with the exception of TE (28.8% for the organocatalyst vs 24.5% for EG).

The contribution analysis also elucidates the degree to which recovery of each component offsets the consumption of EG, energy, and catalyst. For the simpler case of S2, the environmental benefits from recovering BHET far outweigh those of recovering the metals, although metal recovery has a more obvious (yet still minor) contribution in the TE and HNCT categories. The minor relative contribution of metal recovery for both S1 and S2 is due to the fact that only a small amount of metals are embedded in the cards (<1 wt%). For S1, the benefit of recovering CHDM alongside BHET is clear from the absolute and relative data; in many of the highlighted categories (LU, IR, HNCT, and TE), the offsets from CHDM recovery are larger than those of BHET ( −245.6 vs −192.4 m^2^ a crop eq, −278.2 vs −247.7 kBq Co-60 eq, −4529.7 vs −1563.9 kg 1,4-DCB, −5317.0 vs −4478.3 kg 1,4-DCB, respectively; see Table S6).

Coupled with the overall impact assessment shown in Figure 2, the contribution analysis clearly substantiates the overall benefit of recovering CHDM and EG. The ∼67% reduction in GWP from S2 to S1 is achieved in large part by recovering CHDM and therefore offsetting emissions from its synthesis (Figures 2 – 3 and S8–S9). The stark differences in TE and HNCT between S1 and S2 can also be attributed to the recovery of CHDM and EG (Figures 2 and 3). The CHDM and EG recovery also offers slight benefits in the categories of IR and LU, although not enough to offset the positive contributions from the added electricity needed. Nonetheless, these LCA findings strongly suggest that designing a system that recovers all four components (metals, BHET, EG, and CHDM) is more sustainable and generally results in lower environmental impact than a system where only BHET and metals are recovered.

After identifying the key environmental impact drivers, we further investigated the influence of water usage during the chemical recycling process. A large excess of water is used to purify the BHET obtained from depolymerization, as BHET is soluble in hot water and precipitates as relatively pure white crystals on cooling. In the above results, we employed the same mass ratio of water to PET-G cards as was used in our experimental work (3:1).^23^ BHET of higher purity can be obtained by increasing the amount of water used, albeit at the expense of more energy consumption in the evaporation stage. However, the environmental consequences of this sensitivity analysis have been largely overlooked in most studies, with a few exceptions.^36,38^

**Figure 4:**
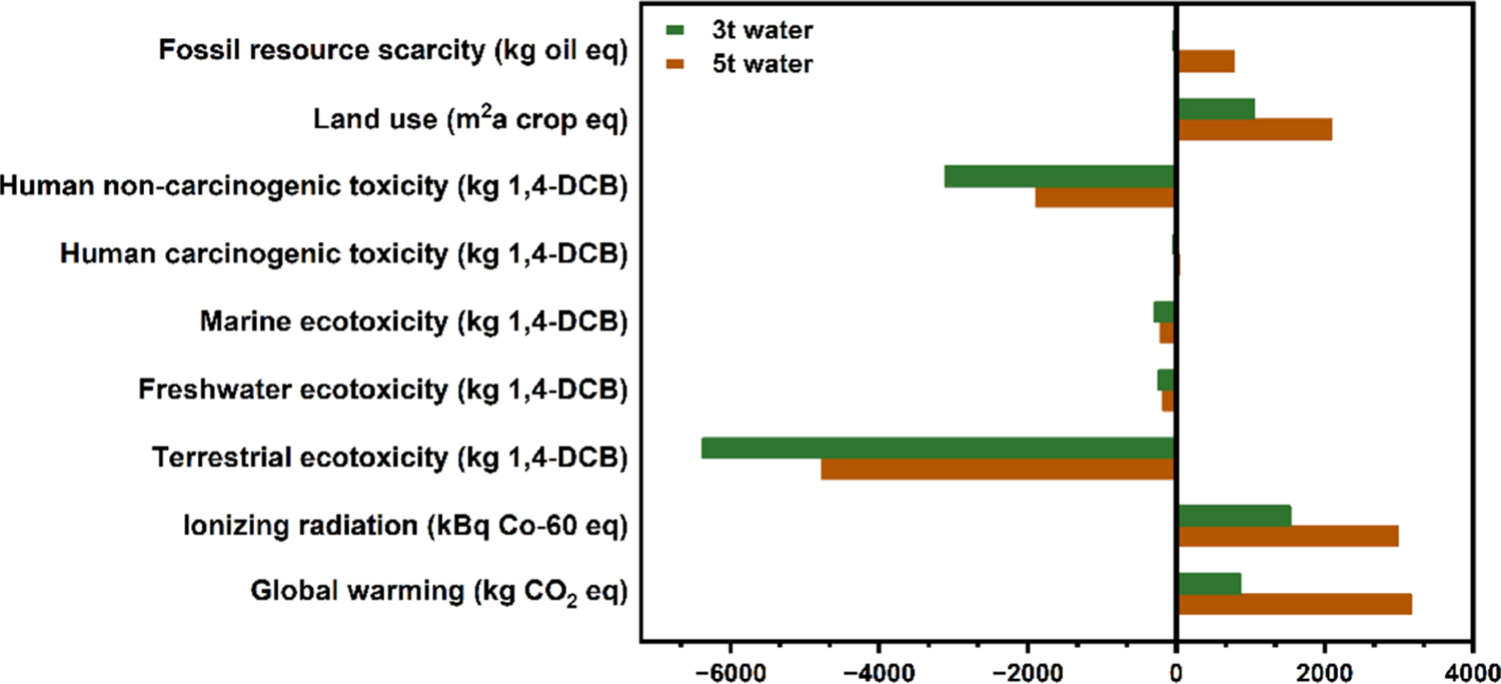
Sensitivity analysis of the overall life cycle impact scores for chemical recycling of payment cards with varying amounts of water during the purification step: 3 tonnes of water (depicted in green) versus 5 tonnes of water (depicted in orange).

To assess the sensitivity of the results to water usage, we increased this parameter in the model for S1 from our previous value of 3 tonnes (i.e., 3 times the mass of PET-G cards) to 5 tonnes. Literature data were used to predict the effect of this change on purity and yield.^39,40^ The revised LCA results showed that increased water usage for BHET purification negatively impacted all impact categories (Figure 4). More specifically, the GWP increased by ∼266%, the IR increased by ∼95%, and LU increased by ∼98%. Furthermore, a complete reversal of the environmental impact was observed in the categories of FRS (−37.8 to 777.6 kg oil equiv) and human carcinogenic toxicity (−38.7 to 51.8 kg 1,4-DCB). These changes in the environmental footprint were primarily caused by the energy required for water recovery through evaporation. To reduce energy consumption in the solvent recovery stage, we further explored the use of low boiling point organic solvents for BHET purification. However, experimental tests with methanol and ethyl acetate demonstrated that the purity of the final product was compromised when using these solvents (Figures S13 and S14).

### Techno-Economic Analysis

In the UK, waste management facility gate fees for different waste treatment, recovery, and disposal options are regularly reported by local authorities. In 2021, the Waste and Resources Action Program (WRAP) conducted a survey to examine these fees and other facility details. According to the survey, the median average gate fees (including transport) for waste sent to a nonhazardous landfill facility was £83/tonne, but gate fees varied widely depending on location, ranging from £15 to £150 per tonne. Moreover, the landfill tax for 2021/2022 was set at £96.7/tonne.^41^ Based on this information, we estimate that the total annual cost of landfill disposal for payment cards (assuming 1 tonne/day) would be between £40,771 and £90,046.

The success of plastic card chemical recycling relies on capital investment, the efficiency of the process, and the selective recovery of depolymerized products. The process must be cost-effective, and the reclaimed products must be of high quality to offset the incurred expenses.^42^ To assess the economic feasibility of the conceptual system, we conducted a preliminary TEA based on Aspen Plus modeling (Figures S16 and S17) and experimental data reported in the literature. As the cost of the reactor was not factored into the automatic estimation in APEA, we manually adjusted the total capital and operating costs to account for all equipment costs (including the reactor) based on the installation costs calculated from APEA.

**Figure 5:**
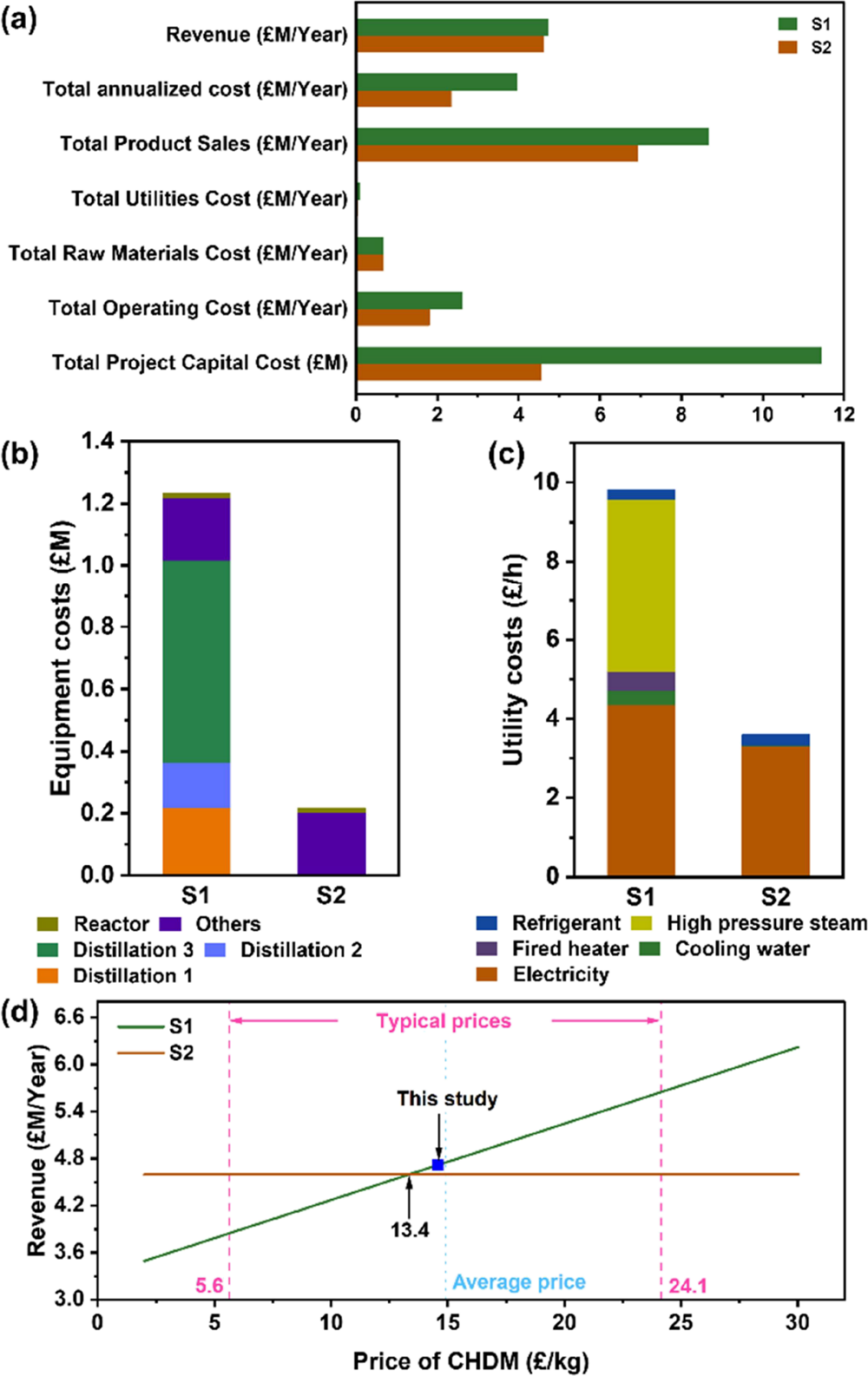
(a) Techno-economic analysis, (b) equipment costs breakdown analysis, (c) utility costs breakdown analysis, and (d) revenue versus price variation of CHDM of S1 and S2. High-pressure steam is used in distillation column 1 for the evaporation of water, and a fired heater is used in columns 2 and 3 for the recovery of ethylene glycol and CHDM. CHDM: 1,4-cyclohexanedimethanol.

The primary distinguishing factor between the two scenarios is the capital cost: the prediction for S1 is 1.5 times greater than that for S2 (Figure 5a). This discrepancy is attributed to the high capital costs of manufacturing the additional high-temperature distillation columns, particularly the one utilized for CHDM recovery (“Distillation 3” in Figure 5b). Compared with S2, the evaporation of a large amount of water—powered by high-pressure steam—led to 173% higher utility costs in S1 (Figure 5c), consequently resulting in 45% higher total operating costs. In contrast, the focus on recovering only metals and BHET in S2 negates the need for distillation columns and thus significantly reduces both capital and operating costs. BHET and CHDM are crucial chemical building blocks utilized in the production of high-value polyester markets with growing demand. The revenue generated from material sales compensates for the incurred capital and operating costs.

The total annualized costs of S1 and S2 amounted to 3.94 £M and 2.33 £M, respectively. Although the separation of all components in S1 resulted in an annual cost that is 1.61 £M higher than that of S2, its annual product sales are 1.73 £M higher, leading to marginally higher revenue for S1 when compared to S2. The additional revenue generated in S1 is largely attributed to the sale of CHDM, which is subject to considerable market volatility. Despite the relatively small quantity of CHDM that can be extracted from the depolymerized cards, it raises concerns about the economic viability of recovering this material, as it necessitates substantial capital investment. To address this issue, we conducted a sensitivity analysis, which revealed that a trade-off between separation costs and product revenue becomes apparent when the market price of CHDM surpasses £13.4/kg (Figure 5d). Discounted cash flow analysis showed that the cash flow positive point for S1 would be achieved after approximately three to five years, assuming a discount rate of 6–9%. However, the profitability of S1 would diminish when the discount rate exceeds 10%. On the other hand, S2 becomes profitable after only two years, within a discount rate range of 6–12%.

When the options of landfill, S1, and S2 for end-of-life management are compared, it is more economically feasible to recover only metals and BHET from the depolymerized cards, even though this approach leads to environmental damage. However, once sufficient profits have been generated and the discount rate is below 10%, it becomes advantageous to recover the other products from the depolymerized system to achieve the net-zero target while retaining profitability. Based on the stated assumptions and current product yields, the techno-economic evaluation of the chemical recycling of payment cards demonstrates a favorable outcome.

### Limitations and Recommendations

The present study has several limitations that warrant discussion. First, the chemical recycling and purification system was solely based on laboratory-scale experiments and process modeling, which constrains their applicability to commercial industrial production settings. Moreover, our study highlights significant variations in the recycling of payment cards, including factors such as the source and composition of PET-G cards, the purity of the input chemicals, the amount of water used for purification, and the desired purity of the recovered products. Thus, conducting LCAs in commercial card production is crucial in order to provide a comprehensive comparison of the associated impacts. This approach has the potential to further enhance the accuracy of the process model and the level of insight that it offers. While our study did not explicitly address the uncertainties stemming from truncation errors within the mass-balance approach, we acknowledge that such errors could potentially influence the final conclusions, thereby introducing a degree of uncertainty into our study.

From an environmental and economic perspective, it is essential to enhance process design and adopt more energy-efficient infrastructure to minimize energy loss during the chemical recycling process. The amount of water utilized in BHET purification is an especially uncertain parameter because of significant variations in the published literature concerning BHET purification as well as the different ratios of BHET oligomers that emerge after polyester depolymerization. The evaporation of a large amount of water is the most energy-intensive step in the purification process. Therefore, optimizing the energy efficiency of Aspen process modeling could considerably reduce energy demand by implementing heat exchanger network synthesis, which considers the trade-off between the number of heat exchangers, the total heat exchanger area, and energy consumption.

In order to further reduce the negative environmental impacts, a shift from fossil-derived energy toward renewable energy sources may be a viable solution, as they do not depend on finite resources. Advances in technology and economics of scale have resulted in a significant reduction in the cost of renewable energy, while fossil fuels have become increasingly expensive due to stricter regulatory measures, depletion of resources, and rising demand. Consequently, the deployment of renewable energy sources can bring substantial economic benefits in the long term.^28^ Overall, these transitions can lead to a reduced energy consumption and significant reductions in the environmental footprint, rendering the chemical recycling process more sustainable.

The comparison of the two scenarios showed that reclaiming all depolymerized products and recycling solvents used in the system is environmentally beneficial. However, this benefit comes at an economic cost. For small- and medium-sized enterprises (SMEs), it may be more economically feasible at the initial stage to recover the primary components (i.e., BHET and metals) from the depolymerized products and dispose of the remaining materials. Once the payout period has been reached, the focus can then be shifted toward building the necessary infrastructure to recover other products, such as CHDM and solvents. This additional recovery process can help to reduce the environmental impacts associated with the chemical recycling process. Furthermore, both scenarios revealed significant environmental impact of the organocatalyst, despite it being used in such small quantities (∼1 wt%). Much of this environmental burden is derived from its synthesis, which highlights the need to design alternatives with similar catalytic efficiency and tolerance to the ambient atmosphere but with fewer steps and less reliance on rare earth metals during synthesis.

One of the uncertainties regarding the potential transition to chemically recyclable plastics is the market dynamics of the recovered depolymerization products. In this case, the key drivers (besides metals) will be demanded for BHET and CHDM. At present, BHET is an intermediate chemical in PET production, and thus, the market price used in this study may not be reflective of the need from the plastics sector; therefore, additional sensitivity analyses on the market price of BHET would be beneficial in determining to what extent the economics of each scenario may be impacted. As both scenarios recover similar amounts of BHET (and metals), we focused our sensitivity analysis on the CHDM market price. Although we anticipate that the increasing adoption of PET-G payment cards (and other PET-G products) will drive demand for CHDM, it is important to acknowledge that further market research and analysis are necessary to validate the assumption that all byproducts of CHDM can be successfully sold. The feasibility of this assumption may change with evolving recycling infrastructure and market dynamics, requiring ongoing monitoring and evaluation for accurate economic assessments. Finally, our present investigation deliberately omits the pursuit of catalyst recovery, despite its significance in the realm of catalytic chemical recycling strategies. This strategic decision was driven by the overarching objective of our study: the isolation and refinement of the primary constituents: BHET, CHDM, and metals. The endeavor to reclaim the DBU catalyst, particularly given its nominal loading (1 wt%), would likely entail a series of energetically demanding separation and purification procedures. These intricacies, while requisite, could significantly augment the overall cost of the industrial-scale chemical recycling process and also potentially induce oxidative degradation of the organocatalyst. Future experimental work and modeling will be carried out to evaluate this possibility and the potential trade-offs between DBU recovery and overall process economics.

Finally, the economics of both scenarios will be dependent on two factors that were also considered out of scope for this initial study: the cost of cards as raw materials and the costs of collection and sorting. These are additional important contributors that would skew the numerical values from the TEA presented herein, although we suspect the trends gleaned from both scenarios would remain. Fortunately, pilot schemes are already underway in the UK^37^ that will ultimately provide data to refine this TEA by addressing these factors. This is especially important for collection costs, as they are heavily dependent on region, method, and market conditions.^43,44^

## Conclusions

In this study, we conducted a comprehensive LCA and TEA to evaluate the environmental and economic impacts of the chemical depolymerization of plastic PET-G payment cards. Our analysis compared two scenarios: recovery of metals, water, EG, BHET, and CHDM (S1) or recovery of only metals and BHET (S2). We found that S1 gives greater environmental benefits, while S2 is more economically feasible.

Importantly, S1 yielded significant reductions (relative to S2) in environmental impacts across several key categories including GWP, TE, and HNCT. These benefits are largely attributed to the contributions gained from CHDM and EG recovery, which easily offset the impacts of added energy needed for distillation/evaporation. The only two categories in which S2 has a lower environmental burden than S1 are IR and LU due to the increased amount of electricity needed for S1.

TEA revealed that S1 generates higher revenue due to the sale of recovered CHDM, despite the higher capital costs required. However, it is only economically feasible to recover all components in S1 when the market price of CHDM exceeds £13.4/kg, which may necessitate an increase in the global PET-G market share. Currently, it is more economically feasible to recover only metals and BHET from depolymerized cards, but it becomes advantageous to recover other products once profits have been generated, and the discount rate is below 10%.

Our study highlights the importance of considering the environmental and economic impacts of different components and processes in the chemical recycling of PET-G multimaterials to develop more sustainable and efficient recycling practices. To transition toward a sustainable, resource-efficient, and circular economy model for plastic cards and other multimaterials, it is crucial to revamp the design and production process and redefine what is achievable through recycling. The proposed chemical depolymerization of plastic cards reduces the dependence on fossil fuels and enables the closure of the materials loop, enabling a potential circular economy for complex multimaterials.

## Supporting Information Available

Composition and sorting of payment cards; life cycle assessment; techno-economic analysis; and Aspen Plus process models (PDF)

## References

[ref1] Xu Z.; Pan F.; Sun M.; Xu J.; Munyaneza N. E.; Croft Z. L.; Cai G. G.; Liu G. Cascade degradation and upcycling of polystyrene waste to high-value chemicals. Proc. Natl. Acad. Sci. U.S.A. 2022, 119 (34), e2203346119 10.1073/pnas.2203346119. 35969757 PMC9407675

[ref2] Rahimi A.; García J. M. Chemical recycling of waste plastics for new materials production. Nat. Rev. Chem. 2017, 1 (6), 0046 10.1038/s41570-017-0046.

[ref3] Geyer R.; Jambeck J. R.; Law K. L. Production, use, and fate of all plastics ever made. Sci. Adv. 2017, 3 (7), e1700782 10.1126/sciadv.1700782. 28776036 PMC5517107

[ref4] Wu X.; Galkin M. V.; Stern T.; Sun Z.; Barta K. Fully lignocellulose-based PET analogues for the circular economy. Nat. Commun. 2022, 13 (1), 3376 10.1038/s41467-022-30735-4. 35697677 PMC9192716

[ref5] Chamas A.; Moon H.; Zheng J.; Qiu Y.; Tabassum T.; Jang J. H.; Abu-omar M.; Scott S. L.; Suh S. Degradation rates of plastics in the environment. ACS Sustain. Chem. Eng. 2020, 8 (9), 3494–3511. 10.1021/acssuschemeng.9b06635.

[ref6] Yuan X.; Wang X.; Sarkar B.; Ok Y. S. The COVID-19 pandemic necessitates a shift to a plastic circular economy. Nat. Rev. Earth Environ. 2021, 2 (10), 659–660. 10.1038/s43017-021-00223-2. 34604789 PMC8475463

[ref7] Bucknall D. G. Plastics as a materials system in a circular economy. Philos. Trans. R. Soc. A 2020, 378 (2176), 20190268 10.1098/rsta.2019.0268. 32623994

[ref8] Soares C. T. d. M.; Ek M.; Östmark E.; Gällstedt M.; Karlsson S. Recycling of multi-material multilayer plastic packaging: Current trends and future scenarios. Resour. Conserv. Recycl. 2022, 176, 105905 10.1016/j.resconrec.2021.105905.

[ref9] Anshassi M.; Townsend T. G. The hidden economic and environmental costs of eliminating kerb-side recycling. Nat. Sustain. 2023, 6, 919–928. 10.1038/s41893-023-01122-8.

[ref10] Lange J. Managing Plastic Waste-Sorting, Recycling, Disposal, and Product Redesign. ACS Sustain. Chem. Eng. 2021, 9 (47), 15722–15738. 10.1021/acssuschemeng.1c05013.

[ref11] Borrelle S. B.; Ringma J.; Law K. L.; Monnahan C. C.; Lebreton L.; Mcgivern A.; Murphy E.; Jambeck J.; Leonard G. H.; Hilleary M. A. Predicted growth in plastic waste exceeds efforts to mitigate plastic pollution. Science 2020, 369 (6510), 1515–1518. 10.1126/science.aba3656. 32943526

[ref12] Lisiecki M.; Damgaard A.; Ragaert K.; Astrup T. F. Circular economy initiatives are no guarantee for increased plastic circularity: A framework for the systematic comparison of initiatives. Resour. Conserv. Recycl. 2023, 197, 107072 10.1016/j.resconrec.2023.107072.

[ref13] Oblak P.; Gonzalez-gutierrez J.; Zupančič B.; Aulova A.; Emri I. Processability and mechanical properties of extensively recycled high density polyethylene. Polym. Degrad. Stab. 2015, 114, 133–145. 10.1016/j.polymdegradstab.2015.01.012.

[ref14] Schyns Z. O.; Shaver M. P. Mechanical recycling of packaging plastics: A review. Macromol. Rapid Commun. 2021, 42 (3), 2000415 10.1002/marc.202000415. 33000883

[ref15] Peng Y.; Yang J.; Deng C.; Deng J.; Shen L.; Fu Y. Acetolysis of waste polyethylene terephthalate for upcycling and life-cycle assessment study. Nat. Commun. 2023, 14 (1), 3249 10.1038/s41467-023-38998-1. 37277365 PMC10241940

[ref16] Arroyave A.; Cui S.; Lopez J. C.; Kocen A. L.; Lapointe A. M.; Delferro M.; Coates G. W. Catalytic Chemical Recycling of Post-Consumer Polyethylene. J. Am. Chem. Soc. 2022, 144 (51), 23280–23285. 10.1021/jacs.2c11949. 36524740

[ref17] Garcia J. M. Catalyst: design challenges for the future of plastics recycling. Chem 2016, 1 (6), 813–815. 10.1016/j.chempr.2016.11.003.

[ref18] Haque F. M.; Ishibashi J. S.; Lidston C. A.; Shao H.; Bates F. S.; Chang A. B.; Coates G. W.; Cramer C. J.; Dauenhauer P. J.; Dichtel W. R. Defining the macromolecules of tomorrow through synergistic sustainable polymer research. Chem. Rev. 2022, 122 (6), 6322–6373. 10.1021/acs.chemrev.1c00173. 35133803

[ref19] Chen Y.; Cui Z.; Cui X.; Liu W.; Wang X.; Li X.; Li S. Life cycle assessment of end-of-life treatments of waste plastics in China. Resour. Conserv. Recycl. 2019, 146, 348–357. 10.1016/j.resconrec.2019.03.011.

[ref20] Ahamed A.; Veksha A.; Yin K.; Weerachanchai P.; Giannis A.; Lisak G. Environmental impact assessment of converting flexible packaging plastic waste to pyrolysis oil and multi-walled carbon nanotubes. J. Hazard. Mater. 2020, 390, 121449 10.1016/j.jhazmat.2019.121449. 31630860

[ref21] Singh A.; Rorrer N. A.; Nicholson S. R.; Erickson E.; Desveaux J. S.; Avelino A. F.; Lamers P.; Bhatt A.; Zhang Y.; Avery G. Techno-economic, life-cycle, and socioeconomic impact analysis of enzymatic recycling of poly (ethylene terephthalate). Joule 2021, 5 (9), 2479–2503. 10.1016/j.joule.2021.06.015.

[ref22] Kim H.; Choi J.; Park J.; Won W. Production of a sustainable and renewable biomass-derived monomer: conceptual process design and techno-economic analysis. Green Chem. 2020, 22 (20), 7070–7079. 10.1039/D0GC02258F.

[ref23] Huang P.; Pitcher J.; Mushing A.; Lourenço F.; Shaver M. P. Chemical recycling of multi-materials from glycol-modified poly (ethylene terephthalate). Resour. Conserv. Recycl. 2023, 190, 106854 10.1016/j.resconrec.2022.106854.

[ref24] Wang Z.; Jin Y.; Wang Y.; Tang Z.; Wang S.; Xiao G.; Su H. Cyanamide as a highly efficient organocatalyst for the glycolysis recycling of PET. ACS Sustain. Chem. Eng. 2022, 10 (24), 7965–7973. 10.1021/acssuschemeng.2c01235.

[ref25] Sun Q.; Zheng Y. Y.; Yun L. X.; Wu H.; Liu R. K.; Du J. T.; Gu Y. H.; Shen Z. G.; Wang J. X. Fe_3_O_4_ Nanodispersions as Efficient and Recoverable Magnetic Nanocatalysts for Sustainable PET Glycolysis. ACS Sustain. Chem. Eng. 2023, 11 (19), 7586–7595. 10.1021/acssuschemeng.3c01206.

[ref26] Arias J. J. R.; Thielemans W. Instantaneous hydrolysis of PET bottles: an efficient pathway for the chemical recycling of condensation polymers. Green Chem. 2021, 23 (24), 9945–9956. 10.1039/D1GC02896K.

[ref27] Fukushima K.; Lecuyer J. M.; Wei D. S.; Horn H. W.; Jones G. O.; Al-megren H. A.; Alabdulrahman A. M.; Alsewailem F. D.; Mcneil M. A.; Rice J. E. Advanced chemical recycling of poly (ethylene terephthalate) through organocatalytic aminolysis. Polym. Chem. 2013, 4 (5), 1610–1616. 10.1039/C2PY20793A.

[ref28] Uekert T.; Singh A.; Desveaux J. S.; Ghosh T.; Bhatt A.; Yadav G.; Afzal S.; Walzberg J.; Knauer K. M.; Nicholson S. R. Technical, economic, and environmental comparison of closed-loop recycling technologies for common plastics. ACS Sustain. Chem. Eng. 2023, 11 (3), 965–978. 10.1021/acssuschemeng.2c05497.

[ref29] 29Worch J. C.; Dove A. P. 100th anniversary of macromolecular science viewpoint: Toward catalytic chemical recycling of waste (and future) plastics. ACS Macro Lett. 2020, 9 (11), 1494–1506. 10.1021/acsmacrolett.0c00582. 35617072

[ref30] Standardization, I. O. F. International Standards 14040 and 14044: Environmental Management - Life Cycle Assessment - Requirements and Guidelines; 2006.

[ref31] Trompeta A. F.; Koklioti M. A.; Perivoliotis D. K.; Lynch I.; Charitidis C. A. Towards a holistic environmental impact assessment of carbon nanotube growth through chemical vapour deposition. J. Clean. Prod. 2016, 129, 384–394. 10.1016/j.jclepro.2016.04.044.

[ref32] Yamada Y. M.; Baek H.; Sato T.; Nakao A.; Uozumi Y. Metallically gradated silicon nanowire and palladium nanoparticle composites as robust hydrogenation catalysts. Commun. Chem. 2020, 3 (1), 81 10.1038/s42004-020-0332-z. 36703481 PMC9814402

[ref33] Guo X.; Xin J.; Lu X.; Ren B.; Zhang S. Preparation of 1, 4-cyclohexanedimethanol by selective hydrogenation of a waste PET monomer bis (2-hydroxyethylene terephthalate). RSC Adv. 2015, 5 (1), 485–492. 10.1039/C4RA10783G.

[ref34] Yang K.; An K.; Choi C.; Jin S.; Kim C. Solubility and esterification kinetics of terephthalic acid in ethylene glycol III. The effects of functional groups. J. Appl. Polym. Sci. 1996, 60 (7), 1033–1039. 10.1002/(SICI)1097-4628(19960516)60:7<1033::AID-APP14>3.0.CO;2-1.

[ref35] Bi Z.; Gao H.; Cao H.; Wang X.; Liu C.; Xu Q.; Zhang Z. Preparation of 1,8-diazabicyclo [5.4.0] undec-7-ene. Chinese Patent CN101279973A, 2008.

[ref36] Luo Y.; Selvam E.; Vlachos D. G.; Ierapetritou M. Economic and Environmental Benefits of Modular Microwave-Assisted Polyethylene Terephthalate Depolymerization. ACS Sustain. Chem. Eng. 2023, 11 (10), 4209–4218. 10.1021/acssuschemeng.2c07203.

[ref37] Bhalla A. Shredding a myth about recycling: It’s time to tackle first-use plastic cards. 2023. https://www.mastercard.com/news/perspectives/2023/shredding-a-myth-about-recycling-it-s-time-to-tackle-first-use-plastic-cards/ (accessed Jul 2023).

[ref38] Selvam E.; Luo Y.; Ierapetritou M.; Lobo R. F.; Vlachos D. G. Microwave-assisted depolymerization of PET over heterogeneous catalysts. Catal. Today. 2023, 418, 114124 10.1016/j.cattod.2023.114124.

[ref39] Goh H.; Salmiaton A.; Abdullah N.; Idris A. Time, temperature and amount of distilled water effects on the purity and yield of bis (2-hydroxyethyl) terephthalate purification system. Bull. Chem. React. Eng. 2015, 10 (2), 143–154. 10.9767/bcrec.10.2.7195.143-154.

[ref40] Pilati F.; Toselli M.; Stramigioli C.; Baldi G.; Capra M.; Osella M.; Bavapilone G. Process to prepare bis (2-hydroxyethyl) terephthalate. European Patent EP0723951A11996, 31.

[ref41] Ogden S.; Robb A.; Roberts D.; Palmer G.; Bell O. Comparing the costs of alternative waste treatment options; 2011/22. https://wrap.org.uk/sites/default/files/2022-07/WRAP 2021 – 22 Gate Fees Report FINAL - 23.05.22 clean _ 0.pdf (accessed Mar 2023).

[ref42] Zhou H.; Ren Y.; Li Z.; Xu M.; Wang Y.; Ge R.; Kong X.; Zheng L.; Duan H. Electrocatalytic upcycling of polyethylene terephthalate to commodity chemicals and H_2_ fuel. Nat. Commun. 2021, 12 (1), 4679 10.1038/s41467-021-25048-x. 34404779 PMC8371182

[ref43] Gradus R. H. J. M.; Nillesen P. H. L.; Dijkgraaf E.; Van koppen R. J. A Cost-effectiveness Analysis for Incineration or Recycling of Dutch Household Plastic Waste. Ecol. Econ. 2017, 135, 22–28. 10.1016/j.ecolecon.2016.12.021.

[ref44] Anshassi M.; Townsend T. G. The hidden economic and environmental costs of eliminating kerb-side recycling. Nat. Sustain. 2023, 6 (8), 919–928. 10.1038/s41893-023-01122-8.

