# Supplementary material for: Circularizing PET-G Multimaterials: Life Cycle Assessment and Techno-Economic Analysis
Source: ACS Sustain Chem Eng. 2023 Oct 13;11(42):15328–37. doi: 10.1021/acssuschemeng.3c04047 (PMC10598876; doi:10.1021/acssuschemeng.3c04047)
Supplement: Supplementary file 1 — sc3c04047_si_001.pdf [file sc3c04047_si_001.pdf]

Supporting Information

Circularizing PET-G Multimaterials: Life Cycle Assessment and Techno-Economic Analysis

Peng Huang,[a] Ashiq Ahamed,[b] Ruitao Sun,[c] Guilhem X. De Hoe,[a] Joe Pitcher,[d] Alan Mushing,[d] Fernando Lourenço,[d] and Michael P. Shaver*,[a]

[a]Department of Materials, Henry Royce Institute, The University of Manchester, Manchester, M13 9PL, United Kingdom

[b]Pragmatic Semiconductor Ltd, Cambridge, CB4 0WH, United Kingdom

[c]School of Engineering, The University of Manchester, Manchester, M13 9PL, United Kingdom

[d]Mastercard DigiSec Lab, 5 Booths Park, Chelford Road, Knutsford, WA16 8QZ, U.K.

*E-mail: michael.shaver@manchester.ac.uk

Number of pages: 25

Number of figures: 19

Number of tables: 13

Contents

1. Composition of PET-G payment cards 2

2. Sorting of payment cards 4

3. Life cycle assessment 5

4. Techno-economic analysis 18

5. References 25

1. Composition of PET-G payment cards

Materials: PET-G cards with a dimension of 85.57 mm × 53.97 mm × 0.76 mm were provided by Mastercard. PET-G pellets were supplied by Push Plastic (USA). To remove any moisture, the pellets were dried for 4 h in a vacuum oven at 60 °C and then stored in a desiccator until further use.

Material Characterization: Thermogravimetric analyses (TGA) of the plastic component of the card and PET-G pellets were performed on a TA SDT650 thermogravimetric analyzer. The specimens were heated from ambient temperature to 600 °C under a nitrogen atmosphere (gas purge rate of 50 cm^3/min), at a heating rate of 10 k/min. After which, the sample was cooled down to 300 °C and held for 2 min to equilibrate. Then, the atmosphere was switched to air with the same purge rate of 50 cm^3/min. The sample was finally heated again from 300 to 800 °C, at a heating rate of 10 k/min.

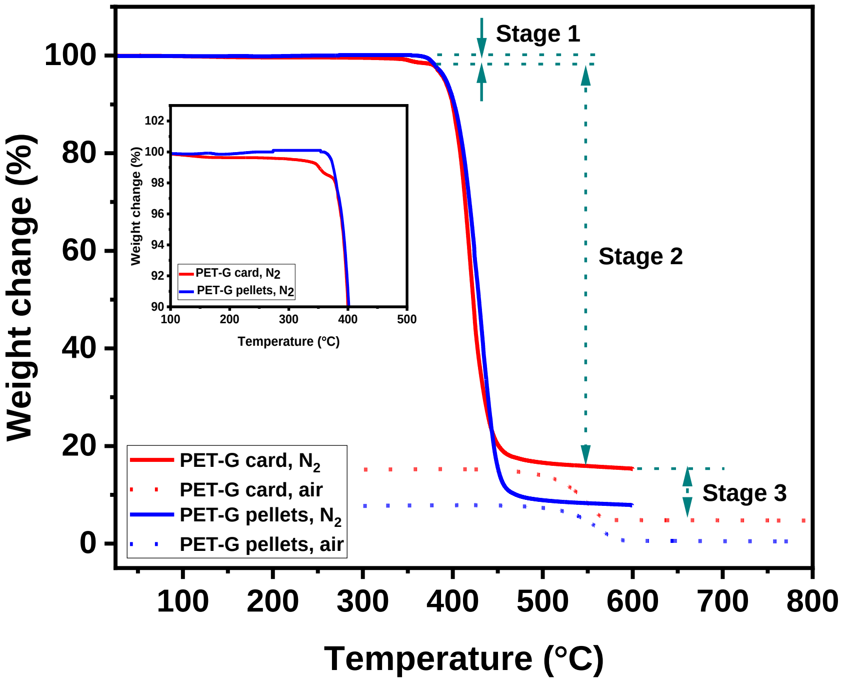

Figure S1. TGA thermograms of PET-G card and PET-G pellets, with an inset showing the mass loss under nitrogen atmosphere during stage 1. After stage 2 the samples were cooled, the gas was switched to air, and the samples were heated again (stage 3).

To determine the composition (*e.g.*, polymer, additives, fillers) of the plastic component of the PET-G cards, TGA experiments were conducted under both inert and reactive atmospheres (Figure S1). Under N_2 atmosphere, the card exhibited a mass loss of 1.6% in the temperature range of 30 – 370 °C, which was attributed to the evaporation of low boiling-point additives (stage 1). This was followed by a mass loss of 82.6%, attributed to the decomposition of the PET-G polymer (stage 2). The subsequent mass loss of 10.5% under air atmosphere was attributed to the combustion of the carbonized polymer (stage 3), and the remaining 5.3% was attributed to the inorganic fillers present in the card. PET-G pellets

were also analyzed under identical conditions for comparison. The differences between the card and the pellets were more clearly visible from the derivative TGA (DTGA) curves (Figure S2); the virgin PET-G pellets only showed the later two stages of degradation, presumably due to the absence of additives.

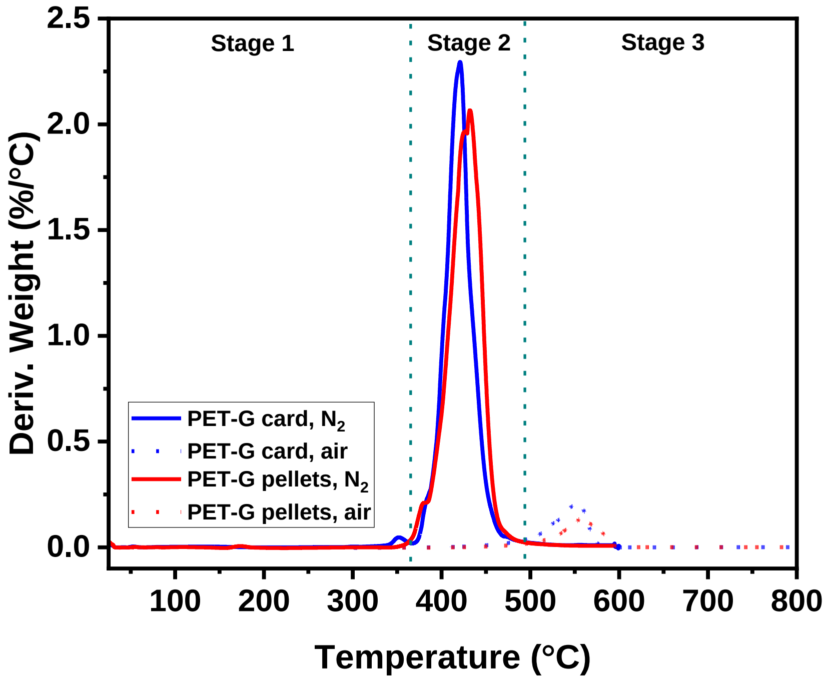

Figure S2. DTGA traces of PET-G card and PET-G pellets.

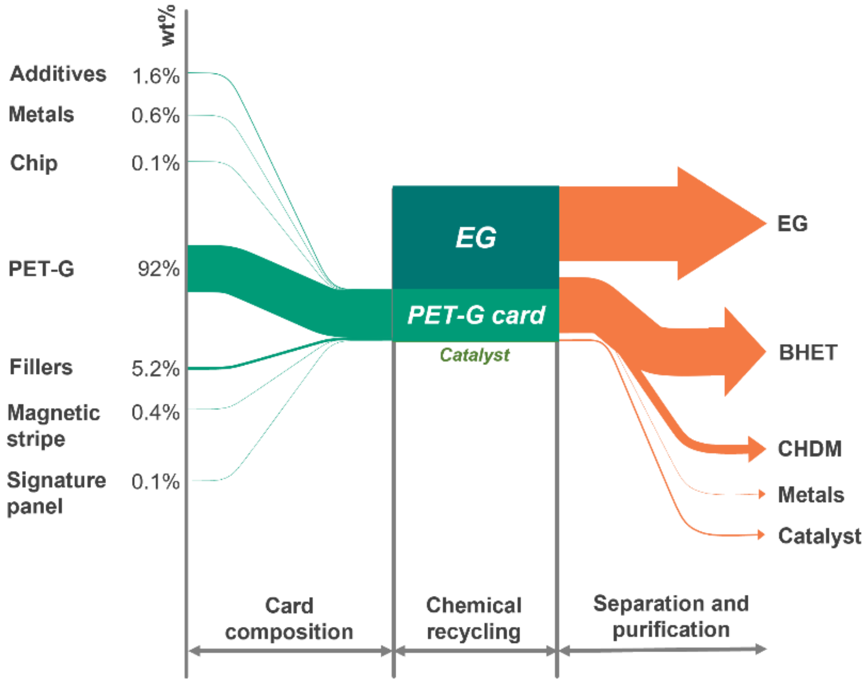

Figure S3. Sankey diagram of the payment card composition and the chemical recycling process. PET-G: glycol-modified poly(ethylene terephthalate). EG: ethylene glycol. BHET: bis(2-hydroxyethyl) terephthalate. CHDM: 1,4-cyclohexanedimethanol.

2. Sorting of payment cards

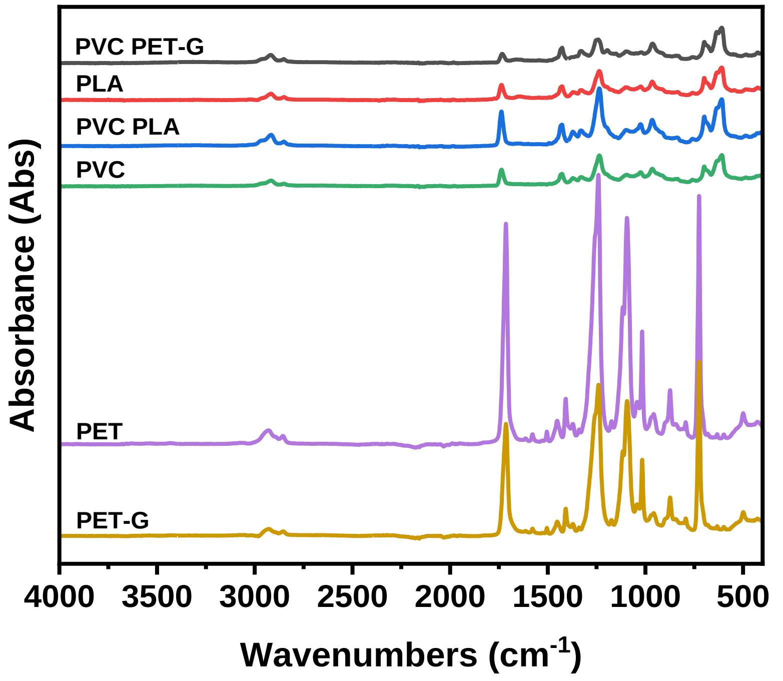

Figure S4. FTIR spectra of typical multi-material payment cards, including those derived from polyvinyl chloride (PVC), polylactic acid (PLA), polyethylene terephthalate (PET), and glycol-modified PET (PET-G). The PLA cards are majority PLA laminated with thin layers of PVC, the PET cards are majority PET laminated with thin layers of PET-G, the PVC/PET-G cards are majority PET-G laminated with PVC, and the PVC/PLA cards are majority PLA laminated with PVC.

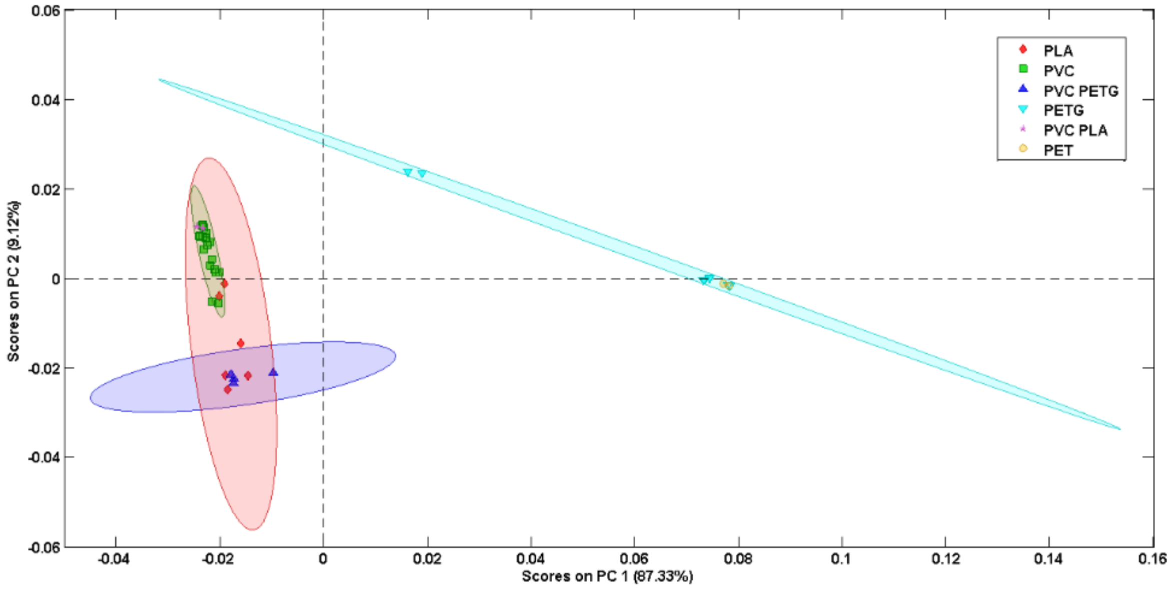

Figure S5. Principal component analysis plot of the 42 payment card samples used in this study. The ATR-IR spectra were pre-processed using a combination of baseline correction (automatic weighted least squares), 1st derivative processing (SavGol, filter width = 7, polynomial order = 2), normalization, and mean-centering. Venetian blinds were used as the cross-validation method (10 splits and 1 sample/split). The 95% confidence ellipse indicates how the payment cards are grouped based on their different plastic composition. The PLA cards are majority PLA laminated with thin layers of PVC, the PET cards are majority PET laminated with thin layers of PET-G, the PVC/PET-G cards are majority PET-G laminated with PVC, and the PVC/PLA cards are majority PLA laminated with PVC.

3. Life cycle assessment

Table S1. LCA basis of the study

**Goal**	The goal is to assess and measure the environmental impacts resulting from the process of depolymerizing waste plastic cards, separating and purifying the depolymerized products, and reclaiming solvents.
**Functional unit**	The functional unit is defined as depolymerizing one tonne of PET-G payment cards per day.
**Scope**	The scope of this investigation begins with the depolymerization process and concludes with the recovery of the recycled products from the system. Any materials that cannot be recovered from the processing steps, such as fillers and additives, are considered to be non-recoverable and will be disposed of as wastewater.
**Target audience**	This study aims to provide valuable insights and information to various stakeholders including waste management professionals in academia, local government officials, policymakers, and industrial sectors. The primary focus is on effective waste management strategies for plastic cards.

The selection of a 1-tonne/day treatment capacity for the plant is influenced by several considerations. While larger plant sizes indeed offer improved economies of scale, the existing market share of chemically recyclable PET-G cards remains relatively modest in comparison to non-recyclable PVC cards. As pilot schemes are already in progress in the UK to facilitate secure and sustainable disposal of expired payment cards, we must account for the transition phase from PVC to PET-G and the establishment of a pragmatic recycling system. This chosen capacity is in alignment with current market dynamics and the focus on conducting proof-of-concept studies.

At a 1-tonne/day capacity, the plant would be equipped to recycle ~200,000 cards daily. Should a scenario of complete collection and recycling of the annual production of 6 billion cards be achieved, approximately 80-90 plants of similar scale could effectively manage the recycling on a global scale. As the recycling infrastructure matures and the circulation of PET-G cards expands, the feasibility of larger treatment capacities is expected to grow from an economic standpoint.

Synthesis of BHET

The BHET used in this study can be prepared from terephthalic acid (TPA) and ethylene glycol (EG), as shown in Scheme S1. The conditions described below were used to evaluate the environmental impact of the BHET preparation (a background process in this study).

The BHET synthesis was conducted following a previously published method.[1] The esterification reaction of TPA and EG was performed with a TPA : EG molar ratio of 1:3, at 210 °C for 610 min until a transparent solution was obtained.

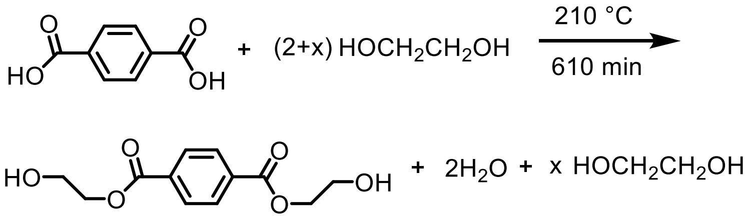

Scheme S1. Synthesis of BHET.[1]

Synthesis of CHDM

The CHDM used in this study can be prepared through the hydrogenation of BHET, as shown in Scheme S2. The conditions described below were used to evaluate the environmental impact of the CHDM preparation (a background process in this study).

Preparation of Cu–Zn/Al_2O_3 catalyst: First, an aqueous solution containing $Cu(NO_3)_2{\cdot}3H_2O$, $Zn(NO_3)2{\cdot}6H_2O$ and $Al(NO_3)_3{\cdot}9H_2O$ with Cu^{2+} : Zn^{2+} : Al^{3+} molar ratio of 5 : 4 : 1 was prepared. Sodium

carbonate was added to the solution with stirring until pH 7 was obtained at 333 K. The resulting precipitate was aged at 333 K for 3 h, then filtered, and washed multiple times with deionized water. The solid sample was dried in an oven at 393 K for 12 h and subsequently calcined at 723 K for 4 h to obtain the oxide. Prior to use, the as-prepared oxide was reduced by H_2 at 573 K.

Hydrogenation of BHET to CHDM: The hydrogenation reactions were performed in a stainless-steel high-pressure reactor with magnetic stirring. The hydrogenation of BHET to CHDM was conducted in two steps (Scheme S2). In the first step, 2.0 g of BHET and 0.1 g of 10% Pd/C catalyst were charged into the reactor, which was then purged with H_2 three times to remove air. The reactor was pressurized to the desired H_2 pressure (5.5 – 7 MPa) and heated to the desired temperature (428 K) at a stirring rate of 400 rpm. After the reaction, the intermediate products, mainly consisting of bis(2-hydroxyethyl) cyclohexane-1,4-dicarboxylate (BHCD), were obtained and used as the raw material for the next step. In the second step, the intermediate products (2.0 g) and Cu-Zn/Al_2O_3 catalysts (0.1 g) were charged into the reactor, which was again purged with H_2 to remove air. The reactor was then pressurized to the desired H_2 pressure (10.5 MPa) and heated to the desired temperature (563 K) under stirring at 400 rpm to obtain the final product, CHDM.[2]

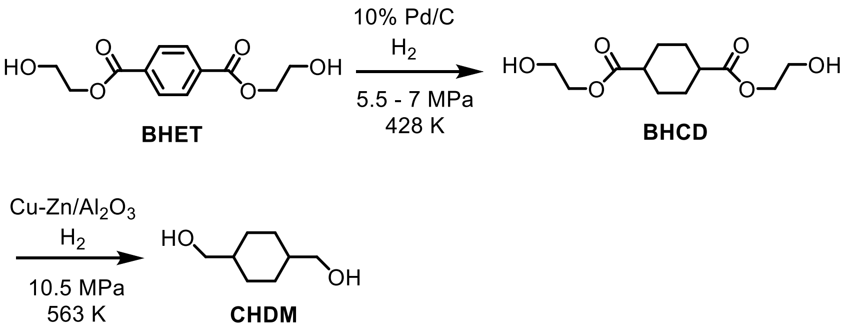

Scheme S2. Synthetic procedure for the hydrogenation of BHET to CHDM.[2]

Table S2. Elementary flows of input and output data for the background process (BHET and CHDM)

	BHET (tonne FU^{-1})		CHDM (tonne FU^{-1})
Input			
Terephthalic acid	0.403	BHET	0.4337
Ethylene glycol	0.301	Pd	0.0022
		C	0.0195
		H_2 gas	0.000014
		Cu	0.0062
		Zn	0.0064
		Al	0.0053
		HNO_3 solution, 50%, v/v	0.1026
Output			
BHET	0.616	CHDM	0.1785
		Ethylene glycol	0.1970
Energy consumption (kWh)	511.28		585

Note: BHET = bis(2-Hydroxyethyl) terephthalate; CHDM = 1,4-cyclohexanedimethanol; FU = functional unit.

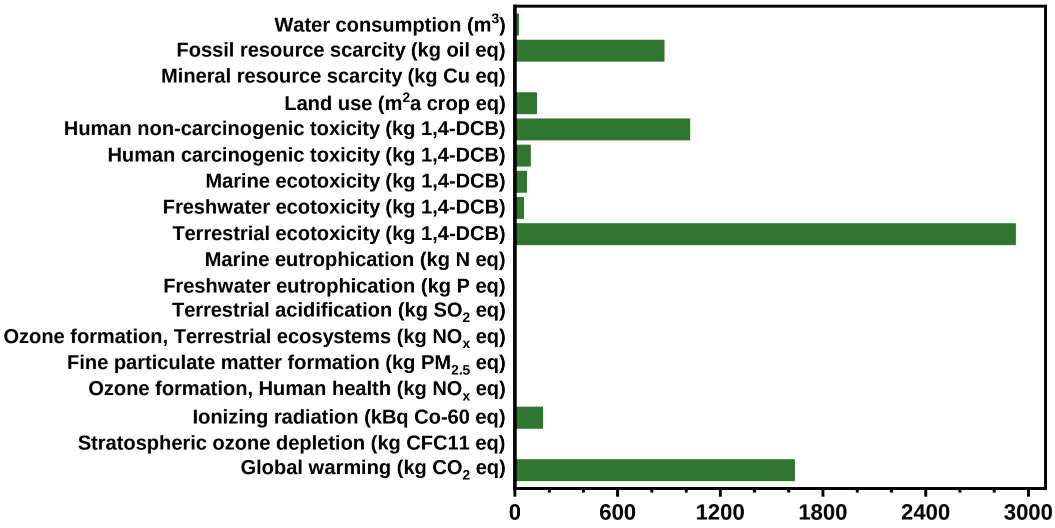

Figure S6. Overall impact assessment of BHET synthesis.

Selvam *et al.* assessed the Global Warming Potential (GWP) for both microwave-assisted waste PET glycolysis and the conventional BHET synthesis process from dimethyl terephthalate (DMT) and EG.[3] In their conventional BHET synthesis, a GWP of 4.37 $kgCO_2eq$/kg BHET was observed, a value 2.5

times higher than our approach utilizing TPA and EG. Notably, if the GWP implications of the BHET production from DMT were considered, the environmental benefits identified in our study would be significantly amplified (2.5 times) beyond our current estimations.

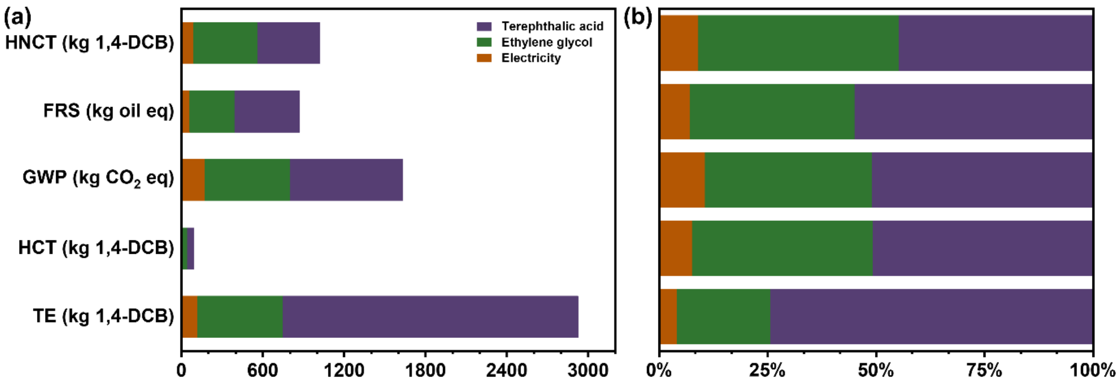

Figure S7. Contribution analysis of BHET synthesis (a) absolute value; (b) percentage.

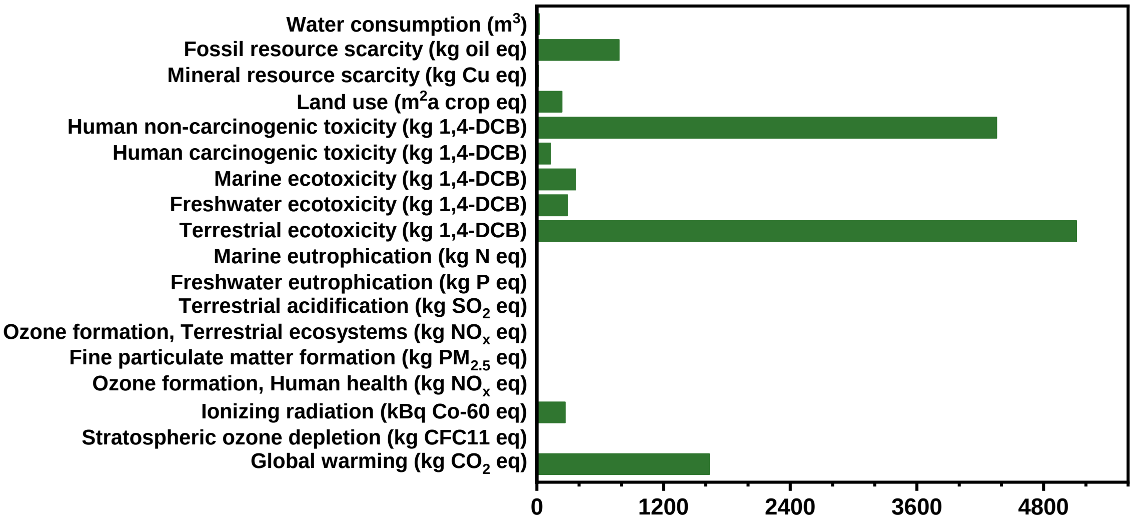

Figure S8. Overall impact assessment of CHDM synthesis.

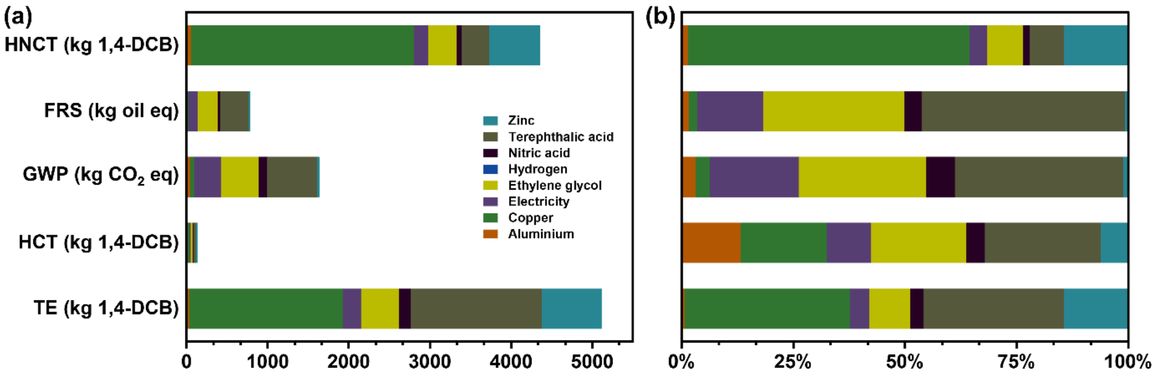

Figure S9. Contribution analysis of CHDM synthesis (a) absolute value; (b) percentage.

Synthesis of DBU

The organocatalyst used for depolymerization in this study (DBU) can be prepared from caprolactam, which is in turn derived from cyclohexanone as shown in Schemes S3 and S4. The conditions described below were used to evaluate the environmental impact of the DBU preparation (a background process in this study).

A 500 mL flask was charged with 70 mL *tert*-butyl alcohol, 100 g caprolactam, and 0.3 g NaOH. The mixture was stirred and heated to 40 °C before adding 50.2 g acrylonitrile. The reaction was maintained at 40–45 °C for 1 h before cooling down to room temperature. The pH was adjusted to 6–7 using H_2SO_4, and the reactants were transferred to a high-pressure reactor containing 10 g Raney Ni catalyst. After adding 30 g ammonia solution, the reactor was pressurized with H_2 gas to 5.0 MPa and heated to 115±5 °C. Upon the completion of the reaction, the solvents were recycled. Then, 3 g *p*-toluenesulfonic acid and 50 mL toluene was added. The temperature was raised to 150–180 °C to complete the dehydration reaction until no water was produced. The solvents were removed to obtain DBU, with a yield of 80% and a caprolactam conversion ratio of 98%.[4]

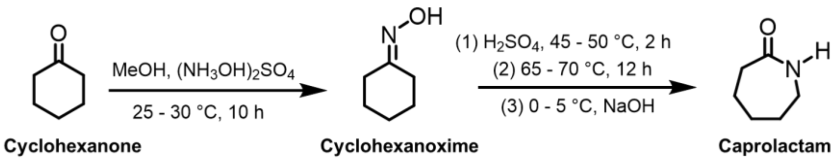

Scheme S3. Synthesis of caprolactam.[4]

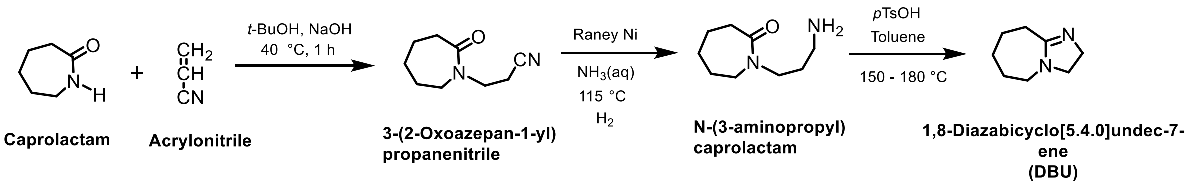

Scheme S4. Synthesis of DBU.[4]

Table S3. Elementary flows of input and output data for the background process (caprolactam and DBU)

	Caprolactam (kg FU^{-1})		DBU (kg FU^{-1})
Input			
Cyclohexanone	24.22	Caprolactam	27.93
Methanol	38.26	Tert-butyl alcohol	15.44
Hydroxylamine sulfate	19.37	Sodium hydroxide	0.08
Sulfuric acid	61.51	Acrylonitrile	14.02
n-Butanol	58.84	Raney Ni	2.79
n-Heptane	32.93	Ammonia solution	8.38
		p-Toluenesulfonic acid	0.84
		toluene	12.15
Output			
Caprolactam	29.40	DBU	36.82
Energy consumption (kWh)	136.6		28.4

Note: DBU = 1,8-diazabicyclo[5.4.0]undec-7-ene. Assuming a 5% weight loss during the handling of chemicals. FU = functional unit.

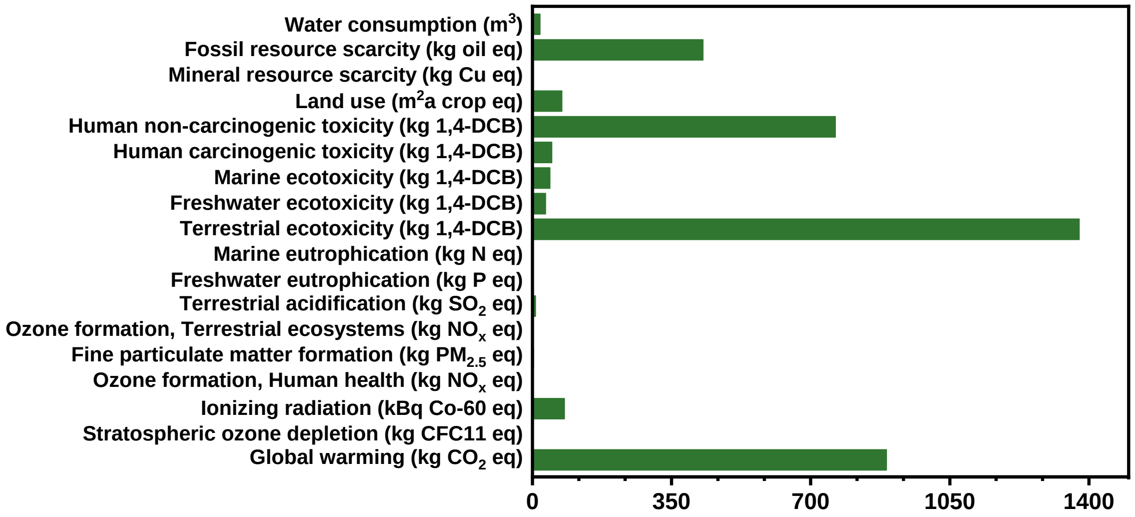

Figure S10. Overall impact assessment of DBU synthesis.

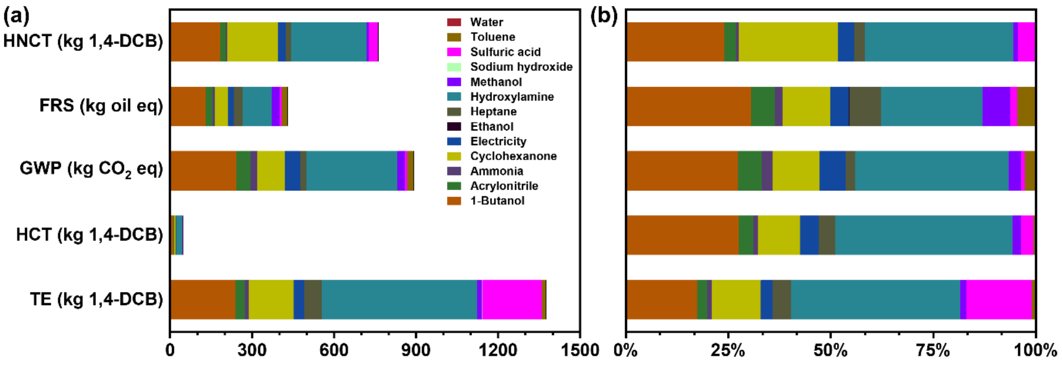

Figure S11. Contribution analysis of DBU synthesis (a) absolute value; (b) percentage.

Table S4. Resources input, output, and energy consumption for the foreground process (Scenario 1, modified to increase water use to 5 tonnes per day)

	**Products/Process**	**(tonne FU^{-1})**
Input	EG	0.542
	Organocatalyst	0.035
	Payment cards	1
	Water	0.505
	Acetone	0.00091
Output	BHET	0.9435
	CHDM	0.1857
	Metals	0.0055
	Wastewater	0.9481
Energy consumption (kJ)	Depolymerization	8.00×10^5
	Distillation of acetone	191.34
	Evaporation of water	3.54×10^7
	Distillation of EG	2.06×10^7
	Distillation of CHDM	6.74×10^4

Note: EG = ethylene glycol; DBU = 1,8-diazabicyclo[5.4.0]undec-7-ene; PET-G = glycol-modified poly(ethylene terephthalate); BHET = bis(2-hydroxyethyl) terephthalate; CHDM = 1,4-cyclohexanedimethanol; FU = functional unit. Energy consumption values were estimated by Aspen Plus.

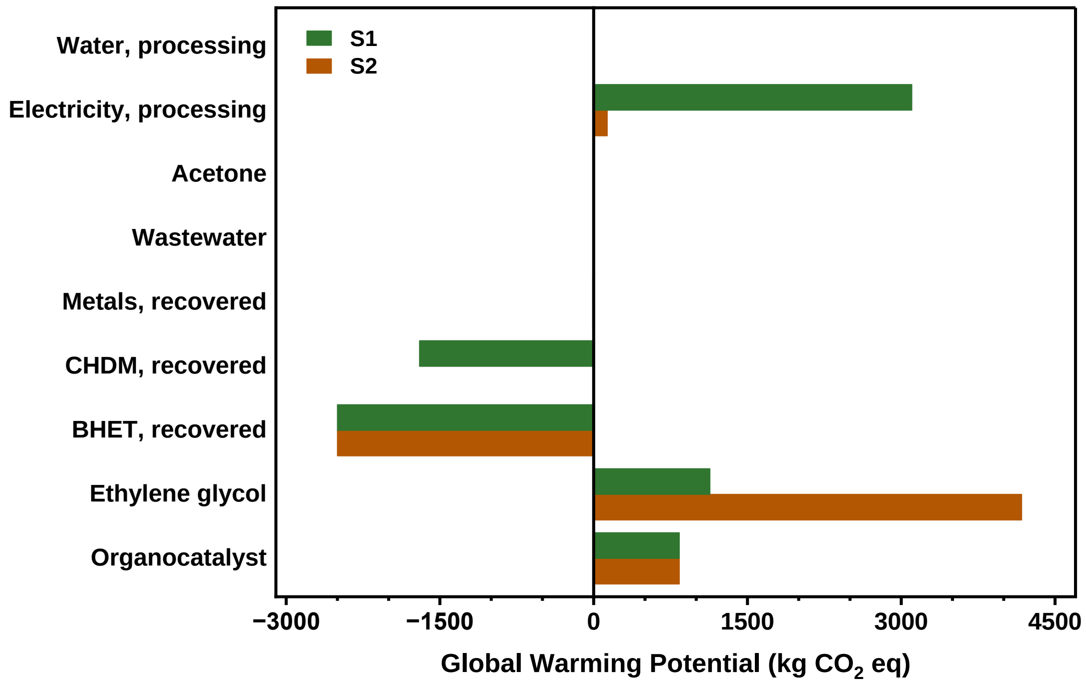

Figure S12. Global warming potential of two scenarios. S1 – recovery of all components; S2 – recovery of metals and BHET.

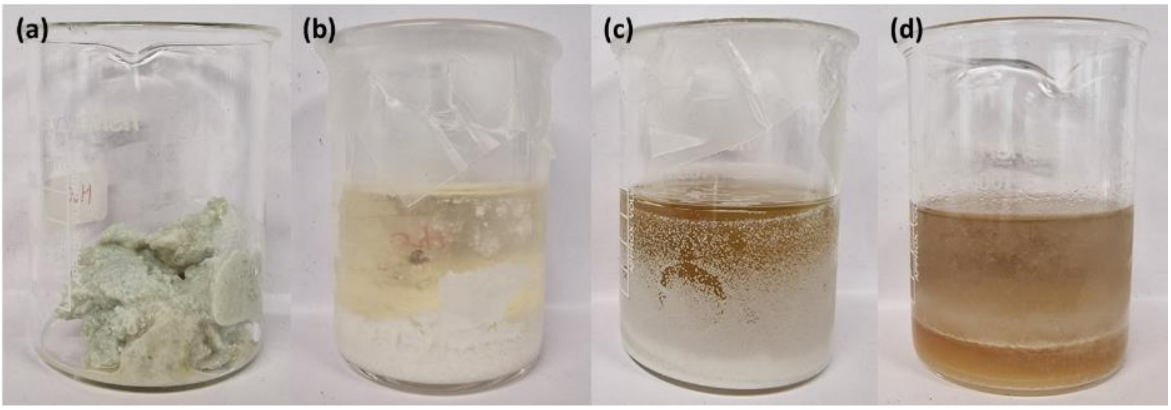

Figure S13. Purification of depolymerized products using different solvents: (a) before purification; Purified in (b) water, (c) methanol, and (d) ethyl acetate.

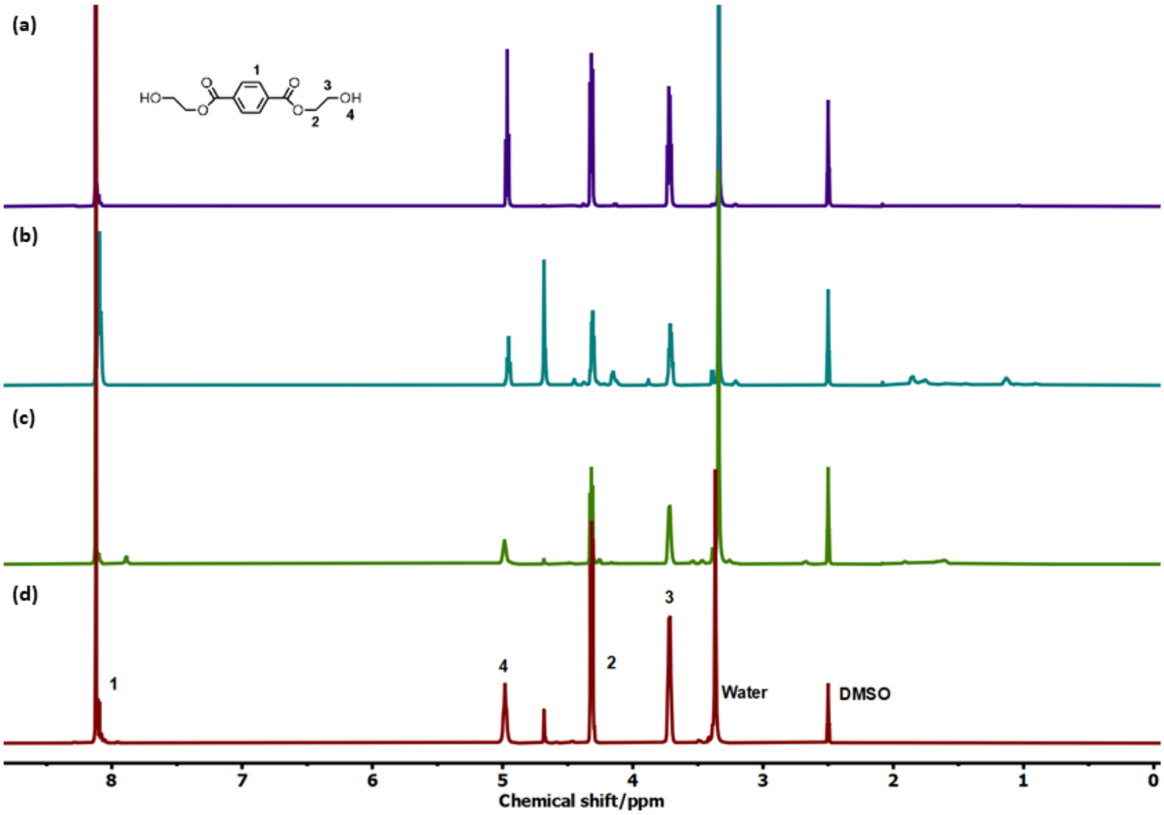

Figure S14. ^{1}H NMR (500 MHz, DMSO-d_6) spectra of purified BHET using different solvents: (a) water, (b) methanol, (c) ethyl acetate, and (d) commercial BHET.

Table S5. Tabulate data for assessing the overall environmental impact of two end-of-life scenarios in the chemical recycling process. S1: Recover all components. S2: Recover only metals and BHET, with the remaining components disposed of as wastewater

	**S1**	**S2**
Water consumption (m^3)	-5.2	40.7
Fossil resource scarcity (kg oil eq)	-37.8	1318.7
Mineral resource scarcity (kg Cu eq)	-17.1	4.7
Land use (m^2a crop eq)	1056.9	81.5
Human non-carcinogenic toxicity (kg 1,4-DCB)	-3118.1	2120.1
Human carcinogenic toxicity (kg 1,4-DCB)	-38.7	160.0
Marine ecotoxicity (kg 1,4-DCB)	-298.4	132.9
Freshwater ecotoxicity (kg 1,4-DCB)	-241.3	103.0
Terrestrial ecotoxicity (kg 1,4-DCB)	-6376.3	-78.4
Marine eutrophication (kg N eq)	0.6	0.7
Freshwater eutrophication (kg P eq)	-0.02	0.9
Terrestrial acidification (kg SO_2 eq)	5.3	13.0
Ozone formation, Terrestrial ecosystems (kg NO_x eq)	0.6	6.2
Fine particulate matter formation (kg $PM_{2.5}$ eq)	1.3	5.5
Ozone formation, Human health (kg NO_x eq)	0.7	5.9
Ionizing radiation (kBq Co-60 eq)	1535.5	78.6
Stratospheric ozone depletion (kg CFC11 eq)	-0.02	-0.01
Global warming (kg CO_2 eq)	864.8	2636.2

Table S6. Tabulate data for the major environmental impact of Scenario 1

	**LU** (m^2**a crop eq)**	**IR (kBq Co-60 eq)**	**HNCT (kg 1,4-DCB)**	**FRS (kg oil eq)**	**GWP (kg** CO_2 **eq)**	**HCT (kg 1,4-DCB)**	**TE (kg 1,4-DCB)**
Water, processing	0.0	0.0	0.1	0.0	0.1	0.0	0.3
Electricity, processing	1402.4	1963.3	1657.4	1100.5	3103.2	122.1	2162.1
Acetone	0.0	0.0	0.4	1.3	2.2	0.1	1.3
Wastewater	0.0	0.0	2.2	0.1	0.4	0.2	1.4
Metals, recovered	-1.5	-0.7	-268.0	-2.3	-10.0	-2.6	-1216.5
CHDM, recovered	-245.6	-278.2	-4529.7	-809.1	-1699.4	-134.7	-5317.0
BHET, recovered	-192.4	-247.7	-1563.9	-1334.0	-2499.8	-137.0	-4478.3
Ethylene glycol	45.4	54.0	851.3	596.7	1133.7	67.1	1133.9
Organocatalyst	48.6	44.7	731.9	409.0	834.5	46.2	1336.5

Note: LU = land use; IR = ionizing radiation; HNCT = human non-carcinogenic toxicity; FRS = fossil resource scarcity; GWP = global warming potential; HCT = human carcinogenic toxicity; TE = terrestrial ecotoxicity.

Table S7. Tabulate data for the major environmental impact of Scenario 2

	LU (m^2a crop eq)	IR (kBq Co-60 eq)	HNCT (kg 1,4-DCB)	FRS (kg oil eq)	GWP (kg CO_2 eq)	HCT (kg 1,4-DCB)	TE (kg 1,4-DCB)
Water, processing	0.1	0.1	1.2	0.3	0.9	0.1	3.1
Electricity, processing	59.4	83.2	70.2	46.6	131.4	5.2	91.6
Acetone	0.0	0.0	0.4	1.3	2.2	0.1	1.3
Wastewater	0.2	0.3	13.1	0.5	2.2	1.0	8.3
Metals, recovered	-1.5	-0.7	-268.0	-2.3	-10.0	-2.6	-1216.5
BHET, recovered	-192.4	-247.7	-1563.9	-1334.0	-2499.8	-137.0	-4478.3
Ethylene glycol	167.0	198.8	3135.2	2197.4	4174.9	246.9	4175.7
Organocatalyst	48.6	44.7	731.9	409.0	834.5	46.2	1336.5

Note: LU = land use; IR = ionizing radiation; HNCT = human non-carcinogenic toxicity; FRS = fossil resource scarcity; GWP = global warming potential; HCT = human carcinogenic toxicity; TE = terrestrial ecotoxicity.

4. Techno-economic analysis

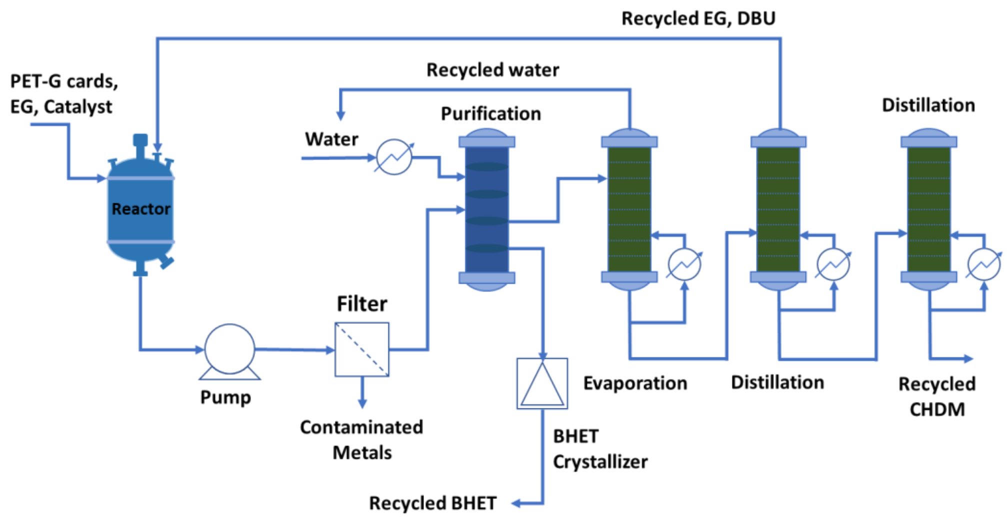

Figure S15. Simplified process flow diagram of the chemical recycling of payment cards.

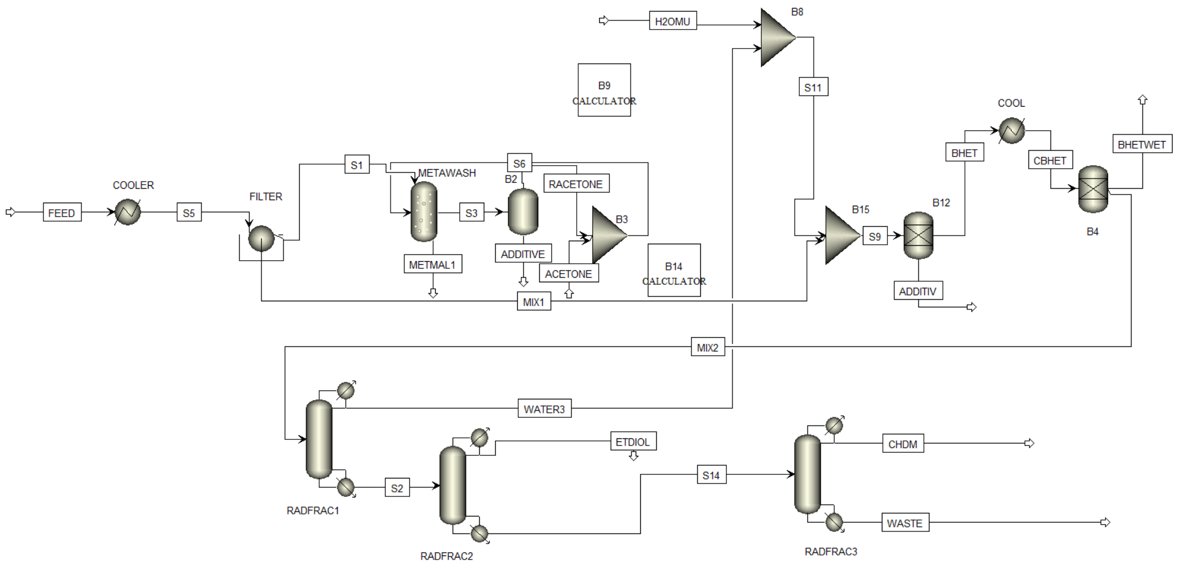

Figure S16. Flowsheet diagram from Aspen Plus model for S1.

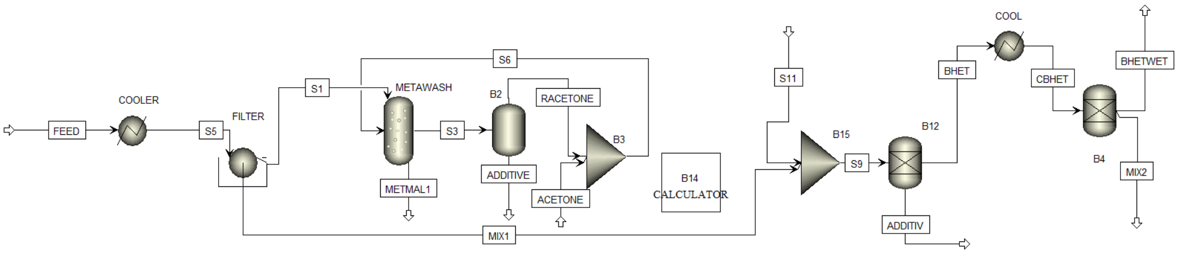

Figure S17. Flowsheet diagram from Aspen Plus model for S2.

Metals, specifically copper, were represented as solid components within the model. Chemicals such as DBU, EG, BHET, and CHDM were modelled as conventional components.

The purification steps for the metal involve several unit operations to ensure effective separation and purification. Initially, metal is separated from the depolymerized products using a filter. The filtered metal is then subjected to a washing process in a Swash unit, where acetone is used as the solvent to remove any impurities and residues.

The crystallizer is represented as a combination of a cooler and a separator. BHET exhibits relatively high solubility in hot water; however, upon cooling, it precipitates from the solution. Subsequently, the white BHET crystals can be readily separated from the liquid phase. The recovery of BHET from recrystallization is estimated based on its solubility parameters in water at various temperatures.[5] At a cooling temperature of 2 °C, our modeling indicates a BHET recovery of 99.4% through recrystallization. Due to the lack of available data on crystallization kinetics for BHET, the capital expenditure estimates pertaining to the crystallizer are exclusively derived from mass and energy balance considerations; we note that crystallization kinetics may impact the required equipment dimensions.

In the RadFrac model, the determination of the tray count initiates with the application of the shortcut model (DSTWU) to acquire preliminary values. These baseline figures subsequently serve as the initial inputs for the RadFrac model. By systematically tailoring the RadFrac model according to predefined design criteria, such as targeted mole recovery and purity, achieved through modulation of reflux ratio and distillate to feed ratio, a sequential process of iterative optimization ensues. This iterative approach culminates in the attainment of the desired operational outcomes.

The application of the NRTL-RK method is specifically directed towards the separation and purification of the depolymerized products, rather than the depolymerization process itself. Detailed and comprehensive experimental data concerning depolymerization, separation, and purification processes can be found in our previous study;[6] the outcomes of these smaller-scale batch experiments served as vital benchmarks for the larger-scale separation and purification process investigated here. Though these processes were simulated under standard conditions and thus an ideal gas approach would have sufficed, we decided to implement the RK equation of state (EOS) to assure the model's future applicability to elevated pressures and non-ideal conditions.

Table S8. Input parameters for the metal recovery process

Unit name	Liquid-to-solid mass ratio	Temperature	Recovery rate	Fraction of liquid to liquid outlet	Fraction of solids to solid outlet
Filter	-	-	-	0.999	1
Swash	0.28	-	-	-	-
Flash	-	80	-	-	-

Table S9. Input parameters for the BHET recovery process

Unit name	Split fraction for water	Split fraction for DBU	Split fraction for EG	Split fraction for BHET	Split fraction for CHDM
Separator (B12)	0.1	0.1	0.1	0.1	0.1
Separator (B4)	0.001	0.001	0.001	0.99	0.001

Table S10. Input parameters for the distillation columns

Column	No. of stages	Feed stage	Pressure (Bar)	Specification
1	32	16	1	Mole purity = 1
				Mole recovery = 0.9999
2	24	12	1	Mole purity = 0.9999
				Mole recovery = 0.999
3	45	23	0.2	-

Note: All columns in the distillation process are equipped with a total condenser and a kettle reboiler. The key variables considered include the reflux ratio and distillate to feed ratio.

Table S11. Summary of results obtained from the distillation columns

Column	Condenser duty (cal/sec)	Reboiler duty (cal/sec)	Utility usage	Reflux ratio	Distillate to feed ratio
1	-71752.1	77325.1	CW usage (tonne/day): 1243.33; HP steam usage (tonne/day): 16.27	3.26	0.86
2	-4591.82	4672.61	CW usage (tonne/day): 79.57; FH steam usage (tonne/day): 2.82	0.33	0.94
3	-293.64	218.80	CW usage (tonne/day): 5.09; FH steam usage (tonne/day): 0.13	0.02	0.96

Note: CW – cooling water; HP – high pressure; FH – fired heater.

Table S12. Parameters used for utility modeling

Utility	Operating conditions	Minimum approach temperature (°C)	Usage	Cost (£/h)	CO_2 energy source efficiency factor
Electricity	-	-	56.20 kW	4.36	0.58
CW	Supply at 20 °C; Return at 25 °C; 1 bar	5	1358.63 (tonne/day)	0.30	-
FH	Supply at 1000 °C; Return at 400 °C	25	2.95 (tonne/day)	0.38	0.85
HP steam	Supply at 250 °C; Return at 249 °C; Vapor fraction = 1	10	16.27 (tonne/day)	3.54	0.85
Refrigerant	Supply at -25 °C; Return at -24 °C	3	374.29 (tonne/day)	0.21	1

Note: CW – cooling water; FH – fired heater; HP – high pressure. The US-EPA-Rule-E9-5711 was used as the CO_2 emission factor data source, and natural gas was selected as the ultimate fuel source.

Table S13. Price of chemicals and products based on market data from September 2022

	Chemicals	Price (£/tonne)	Source
Input	Ethylene glycol	477.9	A
	DBU	1620	B
	H_2O	0.18	C
	Acetone	607.5	D
Output	BHET	16200	E
	CHDM	14580	F
	Metals (copper scrap)	1458	G

Note: Prices were obtained from online sources and published literature (accessed in September, 2022).
A: https://www.alibaba.com/product-detail/Factory-Price-Industrial-Grade-Mono-Ethylene_1600249848319.html?spm=a2700.galleryofferlist.topad_classic.d_title.3ecb6690OlfET9;
B: https://www.alibaba.com/product-detail/China-suppliers-high-quality-DBU-cas_1600342184102.html?spm=a2700.galleryofferlist.normal_offer.d_title.1a6321a40ZHLG7;
C: Liu, W. J.; Xu, Z.; Zhao, D.; Pan, X. Q.; Li, H. C.; Hu, X.; Fan, Z. Y.; Wang, W. K.; Zhao, G. H.; Jin, S. Efficient electrochemical production of glucaric acid and H_2 via glucose electrolysis. *Nat. Commun.* **2020**, *11* (1), 1-11, DOI 10.1038/s41467-019-14157-3.
D: https://hailijia888.en.made-in-china.com/product/rSuQCswgZVRY/China-Best-Price-Acetone-Acetonum-CAS-67-64-1.html.
E: https://www.alibaba.com/product-detail/competition-price-TEREPHTHALIC-ACID-BIS-2_1600444640027.html?spm=a2700.galleryofferlist.normal_offer.d_title.46154a3cyErenG.
F: https://www.alibaba.com/product-detail/CHDM-material-intermediate1-4-Cyclohexanedimethanol-cas_1600338394312.html?spm=a2700.galleryofferlist.normal_offer.d_title.44763f8fU4Nwsk&s=p).
G: https://www.alibaba.com/product-detail/High-Purity-Copper-Wire-Scrap-Cooper_1600629381319.html?spm=a2700.galleryofferlist.normal_offer.d_image.14431fc9d13z3Z).

The capital cost of the reactor is dependent on the processing capacity of the PET-G payment cards. A 2000 L multifunctional stainless steel reactor costs $18,000 (£14575.50, sourced from Alibaba Group Holding Limited). The depolymerization reaction will be conducted three times daily, with each batch requiring the depolymerization of 333.33 kg of payment cards. The costs associated post depolymerization processes, such as separation and purification, were calculated using Aspen Process Economic Analyzer (APEA). Given that the cost of the reactor is not factored into the estimation in APEA, both the total capital and operating costs, including the reactor, were adjusted proportionally based on the installation costs calculated from APEA. The internal rate of return (IRR) is assumed to be 42.5% in the economic analysis.

The installation cost was calculated using the following equation:[7]

$$C = F(\sum C_e) \tag{S1}$$

Here, C is the total plant Inside Battery Limits (ISBL) capital cost, including engineering costs; F is the *Lang factor* (F = 3.63 for processing mixed fluids-solids); C_e is the total delivered cost of the equipment.

The purchased equipment cost is calculated according to the equation below:[7]

$$C = \sum_{i=1}^{i=M} C_{e,i,CS}(1 + \sum_{j=1}^{j=N} f_j) \quad \text{(S2)}$$

Here, $C_{e,i,CS}$ is the cost of purchased equipment *i*; f_j is the installation factor, which includes piping, equipment erection, electrical work, instrumentation and process control, civil engineering work, structures and buildings, lagging, insulation, and painting.

The total annual cost (TAC) was calculated by adding the operating cost (OPEX) and annualized capital cost (CAPEX), which is described by the equation below:

$$ACCR = \frac{DR \times (DR+1)^n}{(DR+1)^n - 1} \quad \text{(S3)}$$

$$TAC = ACCR \times CAPEX + OPEX \quad \text{(S4)}$$

Here, *ACCR* denotes the annualized capital cost ratio; *DR* represents the assumed discount rate of 10%; *n* signifies the estimated period with n = 20. *TAC* represents the total annualized cost.

Discounted cash flow (*DCF*) analysis was performed using the following formula:

$$DCF = \frac{CF_1}{(1+r)^1} + \frac{CF_2}{(1+r)^2} + \cdots + \frac{CF_n}{(1+r)^n} \quad \text{(S5)}$$

Here, CF_i indicates the cash flow period *i (i = 1 — n)*; *r* is the interest rate; and *n* implies the years before the future cash flow occurs.

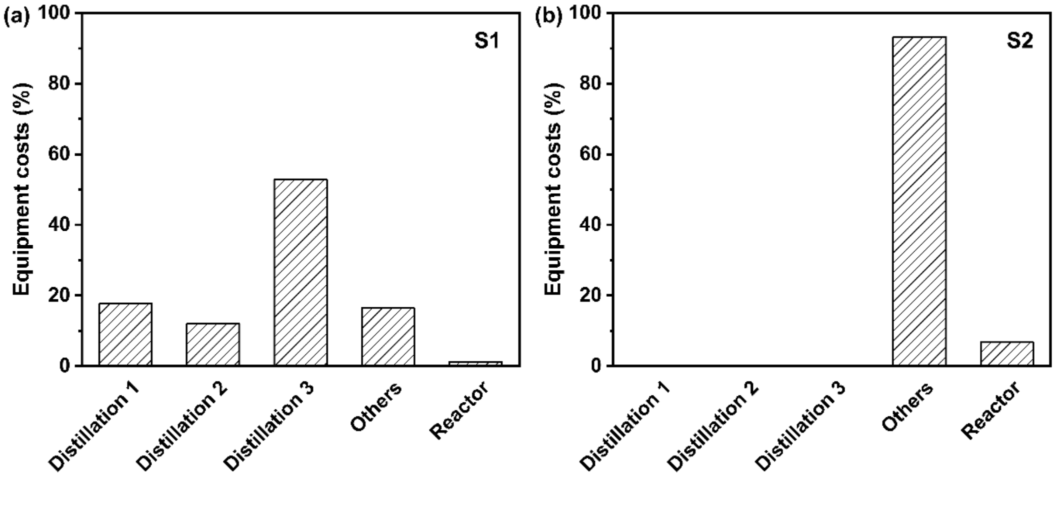

Figure S18. Equipment costs breakdown analysis of (a) S1 and (b) S2 as percentages. The 'Others' category includes single-stage solids washer (SWash), two-outlet flash (Flash2), mixer, separator, and heat exchangers.

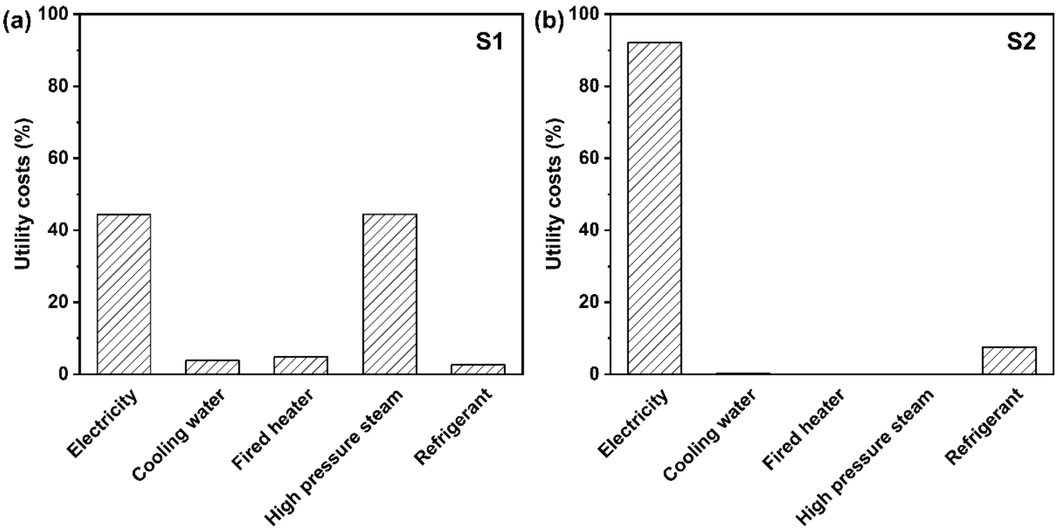

Figure S19. Utility costs breakdown analysis of (a) S1 and (b) S2 as percentages.

5. References

(1) Yang, K.; An, K.; Choi, C.; Jin, S.; Kim, C. Solubility and esterification kinetics of terephthalic acid in ethylene glycol III. The effects of functional groups. *J. Appl. Polym. Sci.* **1996**, *60* (7), 1033-1039, DOI 10.1002/(SICI)1097-4628(19960516)60:7<1033::AID-APP14>3.0.CO;2-1.

(2) Guo, X.; Xin, J.; Lu, X.; Ren, B.; Zhang, S. Preparation of 1, 4-cyclohexanedimethanol by selective hydrogenation of a waste PET monomer bis (2-hydroxyethylene terephthalate). *RSC Adv.* **2015**, *5* (1), 485-492, DOI 10.1039/C4RA10783G.

(3) Selvam, E.; Luo, Y.; Ierapetritou, M.; Lobo, R. F.; Vlachos, D. G. Microwave-assisted depolymerization of PET over heterogeneous catalysts. *Catal. Today.* **2023**, *418*, 114124, DOI 10.1016/j.cattod.2023.114124.

(4) Bi, Z.; Gao, H.; Cao H.; Wang, X.; Liu, C.; Xu, Q.; Zhang, Z. Preparation of 1,8-diazabicyclo[5.4.0]undec-7-ene. Chinese Patent CN101279973A, 2008.

(5) Pilati, F.; Toselli, M.; Stramigioli, C.; Baldi, G.; Capra, M.; Osella, M.; BavaPilone, G. Process to prepare bis (2-hydroxyethyl) terephthalate. European Patent EP0723951A1 1996, *31*.

(6) Huang, P.; Pitcher, J.; Mushing, A.; Lourenço, F.; Shaver, M. P. Chemical recycling of multi-materials from glycol-modified poly (ethylene terephthalate). *Resour. Conserv. Recycl.* **2023**, *190,* 106854, DOI 10.1016/j.resconrec.2022.106854.

(7) Towler, G.; Sinnott, R. *Chemical engineering design: principles, practice and economics of plant and process design*; Butterworth-Heinemann, 2021.
